# Pyruvate kinase M2 in Alzheimer’s disease: from dysregulation to therapeutic inhibition

**DOI:** 10.1093/braincomms/fcag054

**Published:** 2026-02-21

**Authors:** Esmaeel G Gojani, Robert J Sutherland, Majid H Mohajerani

**Affiliations:** Department of Neuroscience, Canadian Centre for Behavioural Neuroscience, University of Lethbridge, Lethbridge, AB, Canada T1K 3M4; Department of Neuroscience, Canadian Centre for Behavioural Neuroscience, University of Lethbridge, Lethbridge, AB, Canada T1K 3M4; Department of Neuroscience, Canadian Centre for Behavioural Neuroscience, University of Lethbridge, Lethbridge, AB, Canada T1K 3M4; Department of Psychiatry, Douglas Mental Health University Institute, McGill University, Montréal, QC, Canada H4H 1R3

**Keywords:** Alzheimer's disease, PKM2, Warburg effect, neuroinflammation, PKM2 modulator

## Abstract

Pyruvate kinase M2 (PKM2) has emerged as a critical regulator of Alzheimer’s disease pathophysiology. This review synthesizes current evidence demonstrating how PKM2 dysregulation contributes to cognitive decline by driving Warburg-like metabolic reprogramming, altering post-translational modifications and modulating protein–protein interactions. These processes collectively impair cell-cycle control, transcriptional regulation and cytoskeletal stability in neuronal cells. We further examine the impact of PKM2 on neuroinflammation, highlighting its context-dependent roles in microglia and astrocytes. In addition, we provide a comprehensive evaluation of natural and synthetic PKM2 modulators with therapeutic potential in Alzheimer’s disease, summarizing their mechanisms and reported outcomes. Clarifying the molecular basis of PKM2-mediated neurodegeneration and rigorously testing these modulators in preclinical models will be essential steps towards developing PKM2-targeted strategies for Alzheimer’s disease intervention.

## Teaser

Ghasemi *et al*. report in this review that the metabolic enzyme pyruvate kinase M2 influences neuronal metabolism, cell-cycle re-entry, cytoskeletal regulation, amyloid-β and tau pathology, neuroinflammation and astrocyte function in Alzheimer’s disease. They conclude that targeting pyruvate kinase M2 may provide promising therapeutic avenues for slowing neurodegeneration.

## Graphical Abstract

**Graphical Abstract:**
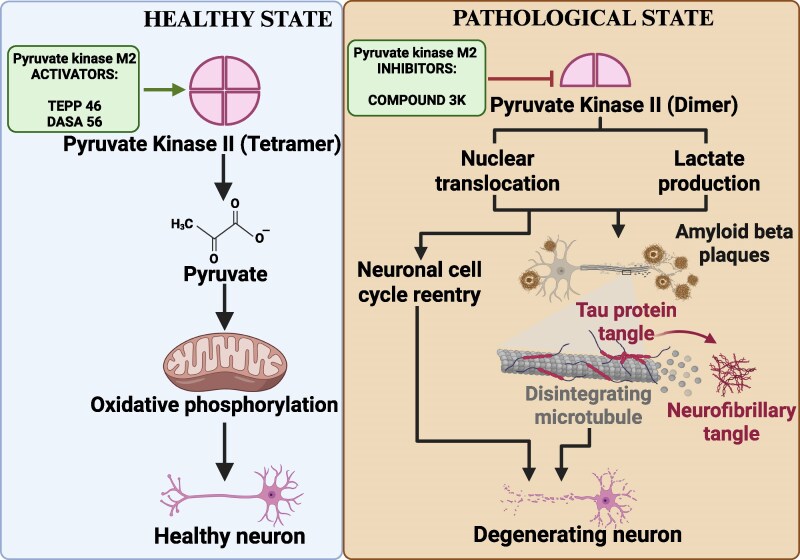

## Introduction

The metabolic demands of various brain cell types differ. Neural stem cells (NSCs; see Supplementary Table 1 for the full list of abbreviations) primarily rely on glycolysis and anabolic pathways for proliferation but require metabolic flexibility for differentiation. Mature neurons, in contrast, depend on oxidative phosphorylation for stability. Oligodendrocytes focus on lipid synthesis for myelin production, while microglia shift from oxidative phosphorylation during rest to glycolysis when activated, supporting proliferation and function.^1^ The reliance of NSCs and activated microglia on glycolysis parallels the metabolic behaviour observed in Alzheimer's disease, where neuronal cells revert to an immature, proliferative-like state, contributing to neurodegeneration.^2,3^ In contrast, healthy neurons primarily depend on oxidative phosphorylation for ATP production due to its high efficiency, generating 30 ATP per glucose molecule. Unlike other cells, they use glycolysis minimally, lacking key enzymes like PFKFB3, making them reliant on mitochondrial energy. Neuronal metabolism is further distinguished by its compartmentalization within individual cells. For instance, while neuronal somata primarily utilize aerobic glycolysis, axon terminals depend on mitochondrial OXPHOS to meet their high energy demands. This spatial distribution of metabolic pathways underscores the delicate balance maintained by pyruvate kinase M2 (PKM2) within neurons, where disruptions in its regulation can destabilize this compartmentalization and contribute to disease progression.^4^ Astrocytes support neurons by supplying lactate via the astrocyte–neuron lactate shuttle, which neurons convert into pyruvate for oxidative phosphorylation.^1,5,6^

Alzheimer's disease is a progressive neurodegenerative disorder and the leading cause of dementia, accounting for 60–80% of all cases. It gradually erodes an individual’s capacity to carry out daily activities, presenting with symptoms such as loss of motivation, mood disturbances, impaired communication, confusion, diminished decision-making and broader behavioural changes.^7–9^ Neuropathologically, Alzheimer's disease is characterized not only by extracellular amyloid-beta (Aβ) plaques and the intracellular Tau neurofibrillary tangles (NFTs),^10^ but also by chronic neuroinflammation,^11,12^ metabolic alterations,^13–15^ neuronal dedifferentiation^16,17^ and synaptic dysfunction,^18,19^ which finally result in cognitive decline.^20^ While amyloid and tau pathologies have dominated Alzheimer's disease research, a growing body of evidence suggests that Alzheimer's disease is fundamentally a disorder of brain metabolism. Neurons, microglia and astrocytes in Alzheimer's disease exhibit marked metabolic dysfunction—including insulin and IGF-1 resistance, oxidative stress and impaired glucose uptake—which are now viewed as major drivers of disease progression.^13,21^ In late-onset Alzheimer's disease, these abnormalities manifest as profound bioenergetic failure, including reduced NAD/NADH ratios, impaired lactate utilization and decreased Krebs cycle efficiency. Although compensatory pathways such as β-oxidation, increased malate–aspartate shuttle activity and oxidative phosphorylation become upregulated, they often fail to restore metabolic homeostasis. Consistent with this, PET imaging reveals hypometabolism in the hippocampus and posterior cingulate cortex—regions critical for memory and cognition. These energetic deficits compromise synaptic function and destabilize neuronal identity, increasing vulnerability to neurodegeneration.^22,23^

The metabolic reprogramming observed in Alzheimer's disease closely mirrors the Warburg effect first described by Otto Warburg in the 1920s, wherein cells favour glycolysis over oxidative phosphorylation despite adequate oxygen availability.^24–26^ This glycolytic bias, normally characteristic of neural progenitor cells and activated microglia, becomes aberrantly re-engaged in mature neurons and glia in Alzheimer's disease.^27,28^ As mitochondrial efficiency declines and glucose utilization becomes impaired, affected brain regions shift towards glycolysis, creating a Warburg-like metabolic state that promotes cellular dedifferentiation and dysfunction. This pathological reprogramming reflects a maladaptive form of plasticity, echoing mechanisms observed in cancer biology.^15,29^

A central driver of this metabolic shift is PKM2, the glycolytic enzyme that converts PEP to pyruvate. While PKM2 normally supports energy production, its role expands dramatically under pathological conditions. When present in its dimeric form, PKM2 can promote processes such as dedifferentiation, neuroinflammation, Aβ plaques and NFT formation.^15,30–32^ This structural shift also alters how glucose is used: instead of supporting efficient mitochondrial oxidation, dimeric PKM2 redirects glucose-derived carbons towards biosynthetic and redox-buffering pathways.^33^ Although this may help maintain short-term cellular stress responses, it ultimately worsens ATP shortages and increases oxidative stress—changes that are particularly damaging to neurons, which rely heavily on robust mitochondrial energy production.^34^

Building on these observations, this review explores how such metabolic alterations shape the progression of Alzheimer's disease, with particular attention to the emergence of Warburg-like reprogramming in vulnerable neuronal populations. We highlight the central role of PKM2 in driving these shifts and examine recent evidence showing how its modulation can influence metabolic efficiency, neuroinflammation and cellular stress responses. By integrating insights from metabolic biology and neurodegeneration, this review outlines how targeting PKM2 may help restore metabolic balance and strengthen neuronal resilience, positioning it as a compelling therapeutic candidate in Alzheimer's disease.

### Consent statement

All human subjects involved in this study provided informed consent prior to participation, or in cases where consent was not required, appropriate ethical approval was obtained.

## PKM isoforms: structure, function

Glycolysis depends on pyruvate kinase (PK) to catalyse the final step of the pathway, converting PEP into pyruvate while generating ATP in an oxygen-independent manner.^35^ Unlike mitochondrial respiration, this oxygen-independent ATP generation allows tissues to maintain energy production under hypoxic conditions.^36^ PK exists as several tissue-specific isoenzymes.^37^ Mammals possess four PK isoforms—PKM1, PKM2, PKR and PKL—each associated with distinct tissues).^38^ PKM2 is the predominant isoform in many adult tissues and is especially abundant in proliferating or metabolically flexible cells, whereas the other isoforms show more restricted distributions: PKM1 is primarily found in muscle, heart and brain; PKL is largely confined to the liver; and PKR is specific to red blood cells.^39,40^

Beyond its classical function in glycolysis, PKM2 plays a key role in metabolic reprogramming, particularly in proliferative and immunologically active contexts. Although expressed at low levels in quiescent cells, PKM2 is strongly upregulated in rapidly dividing tumour cells and activated macrophages, where it facilitates a shift from oxidative phosphorylation to aerobic glycolysis. This Warburg-like adaptation supports both ATP generation and biosynthetic demands required for high cellular activity.^41^

**Figure 1:**
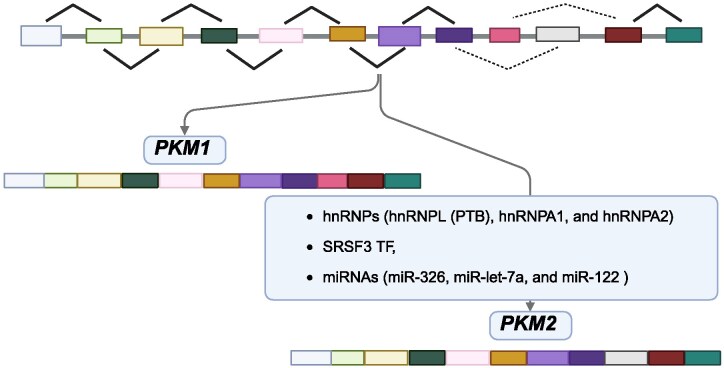
**Alternative splicing of the PKM gene into PKM1 and PKM2 isoforms.** The PKM gene undergoes mutually exclusive splicing of exon 9 or exon 10, producing two isoforms: PKM1, which includes exon 9 and supports oxidative phosphorylation, and PKM2, which includes exon 10 and enables glycolytic and biosynthetic flexibility. Splicing is regulated by hnRNPs (PTBP1, hnRNPA1 and hnRNPA2) that suppress exon 9 inclusion, and by SRSF3, which promotes exon 10 inclusion. MicroRNAs (e.g. miR-122, miR-326 and let-7a) and lncRNAs (e.g. H19 and MEG3) further modulate this process by targeting splicing factors. This regulation determines the PKM1/PKM2 balance in development and disease. PKM, pyruvate kinase; SRSF3, serine/arginine-rich splicing factor 3; TF, transcription factor; hnRNP, heterogeneous nuclear ribonucleoprotein; miRNA, microRNA.

The functional distinction between PKM1 and PKM2 originates from alternative splicing of the same *PKM* gene. While the isoforms share several exons, their unique sequences arise from mutually exclusive exon selection.^42^ The human *PKM* gene spans 32 kb and, similar to its rat counterpart, contains regulatory elements in the 5′-flanking region and conserved intronic sequences that influence tissue-specific splicing and expression.^43^ Exon selection determines the production of each isoform: exon 9 is specific to PKM1, while exon 10 is specific to PKM2. Repression of exon 9 and inclusion of exon 10 lead to the production of PKM2 (Fig. 1).^44,45^

Structurally, mammalian PK is a tetrameric protein composed of identical subunits arranged in a dimer-of-dimers configuration. Each subunit contains an active site and three main domains (A, B and C).^46^ The C domain contains the fructose-1,6-bisphosphate (FBP)-binding pocket, which is particularly important in PKM2, PKL and PKR for activity regulation.^47,48^ This allosteric regulation distinguishes PKM2 from PKM1: while PKM1 forms a stable, constitutively active tetramer, PKM2 can dynamically shift between low-activity dimers and active tetramers in response to FBP binding. The relative balance of these two PKM2 forms is essential for fine-tuning glycolytic flux, allowing cells to adjust energy production according to metabolic demands in both proliferative and differentiated states.^49–52^ In cancer and other proliferative settings, PKM2 dimerization—promoted by post-translational modifications (PTMs) such as phosphorylation, oxidation and acetylation—shifts the enzyme toward its low-activity form, diverting glycolytic intermediates toward biosynthetic pathways to support cell proliferation.^53^

**Figure 2:**
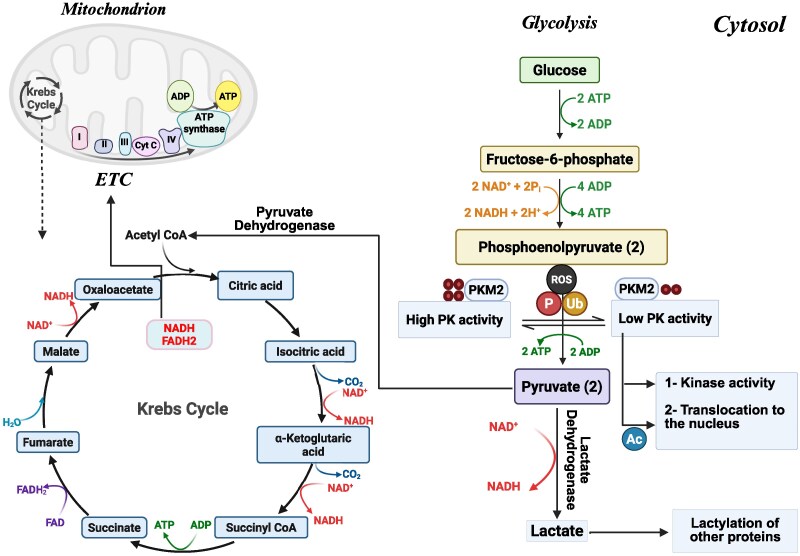
**PKM2 regulation and its role in cellular metabolism.** The figure illustrates how PKM2 integrates glycolysis and mitochondrial metabolism. In its tetrameric form, PKM2 exhibits high enzymatic activity, promoting ATP production and oxidative phosphorylation. In contrast, the dimeric form has low activity, leading to the accumulation of glycolytic intermediates that support biosynthesis and antioxidant defence. Dimeric PKM2 can also translocate to the nucleus, where it regulates gene expression and contributes to PTMs, including lactylation. These metabolic and non-metabolic functions are regulated by phosphorylation, acetylation and ubiquitination, particularly under stress conditions such as hypoxia and oxidative stress, which are relevant to neurodegeneration and cancer. P, phosphorylation; Ub, ubiquitination; ROS, reactive oxygen species; Ac, acetylation; ETC, electron transport chain; OXPHOS, oxidative phosphorylation; Cyt C, cytochrome c; NAD, nicotinamide adenine dinucleotide (oxidized); NADH, nicotinamide adenine dinucleotide (reduced); FADH₂, flavin adenine dinucleotide (reduced); ADP, adenosine diphosphate; ATP, adenosine triphosphate.

Importantly, the dimeric form of PKM2 also exhibits non-metabolic functions, including nuclear translocation, protein kinase activity and gene regulation^33,53,54^ (Fig. 2). In non-proliferative cells such as neurons, these functions become particularly relevant under neurodegenerative conditions like Alzheimer's disease. Neurons from Alzheimer's disease patients exhibit a shift from PKM1 to PKM2, accompanied by enhanced aerobic glycolysis and nuclear translocation of PKM2. This Warburg-like adaptation occurs independently of cell-cycle division and is linked to transcriptional reprogramming, redox imbalance and increased susceptibility to mitochondrial stress.^55^ Together, these findings indicate that PKM2 contributes to Alzheimer's disease pathogenesis by linking metabolic and gene-regulatory networks, highlighting its broader role in neuronal stress responses beyond classical proliferative contexts.

## Regulation of PKM2: signalling pathways, transcriptional control and splicing mechanisms

PKM2 activity is tightly regulated by signalling pathways, transcription factors, PTMs and metabolic cues—particularly in response to hypoxia, inflammation and oxidative stress, key features of Alzheimer's disease pathogenesis.^33^ These regulatory layers collectively determine its oligomeric state, enzymatic activity, nuclear localization and non-canonical functions.

### Transcriptional and splicing mechanism

Transcriptional and splicing mechanisms play a central role in regulating *PKM2* expression. Various transcription factors—including NF-κB,^56^ Oct4^57^ and PPAR-γ^58^—can induce *PKM2* expression in a context-dependent manner, often through upstream signalling pathways such as PKC,^56^ mTOR/AKT^58^ or HIF-1α^59^ stabilization. HIF-1α is especially important in regulating *PKM* gene transcription.^60^ Under hypoxic conditions, stabilized HIF-1α dimerizes with HIF-1β to activate *PKM* transcription via hypoxia response elements. This interaction establishes a feedforward loop, as the resulting PKM2–HIF-1α complex translocates to the nucleus and further enhances the expression of *HIF-1* target genes, reinforcing metabolic reprogramming.^61^ This loop is further modulated by upstream signalling pathways: PI3K/mTOR^62,63^ and AMPK/mTOR^64^ enhance HIF-1α-dependent *PKM2* expression under hypoxic-ischaemic conditions, whereas PTEN counteracts these effects by inhibiting mTOR, thereby reducing PKM2 levels.^65^

Splicing regulation of *PKM* is primarily controlled by hnRNPs—including hnRNPL (PTB), hnRNPA1 and hnRNPA2—which repress exon 9 inclusion, thereby favouring *PKM2* expression.^45,66,67^ Complementing this, the serine/arginine-rich splicing factor SRSF3 promotes exon 10 inclusion, further driving PKM2 isoform production.^68,69^

In addition to splicing factors, non-coding RNAs contribute to fine-tuning *PKM2* expression. For example, miRNAs such as miR-326, miR-let-7a and miR-122 modulate PKM2 indirectly by targeting splicing regulators like PTBP1, shifting the balance from PKM1 towards PKM2.^70–77^ Long non-coding RNAs, including LncRNA-H19 and LncRNA-MEG3, interact with these miRNAs to further refine PKM2 levels, often impacting cellular proliferation and signalling pathways.^78,79^ Taken together, *PKM2* expression is tightly controlled by a coordinated network of splicing factors, miRNAs and lncRNAs, highlighting the complexity of its regulation. This multilayered control, along with associated transcriptional mechanisms, has been comprehensively reviewed by Zhang *et al*.^33,80^

### Post-translational modification

PTMs further refine PKM2 activity and localization. For instance, hydroxylation by PHD3 stabilizes the PKM2–HIF-1α interaction and facilitates PKM2 nuclear accumulation, allowing it to act as a transcriptional coactivator.^81^ Building on this regulatory framework, under conditions of oxidative stress, a hallmark of Alzheimer's disease brains,^82–84^ PKM2 undergoes PTMs that convert it from its active tetrameric form to a less active dimer. For example, phosphorylation at tyrosine 105—often mediated by FGFR1—disrupts the binding of its allosteric activator, FBP, impairing tetramer formation and promoting the low-activity dimeric state.^52,85^ This mechanism can be further potentiated by FGF2, which is elevated in Alzheimer's disease brains,^86,87^ and by cytokine-driven JAK2 activation in pro-inflammatory environments.^52,88,89^ Oxidation of cysteine residues—particularly Cys358 and Cys424—by reactive oxygen species (ROS) similarly disrupts inter-subunit interactions of PKM2, thereby inhibiting tetramer formation and shifting the equilibrium towards the dimeric form. This dimeric state supports antioxidant defence mechanisms and anabolic metabolism under conditions of redox stress.^90^ Acetylation also modulates PKM2 nuclear functions: p300-mediated acetylation at Lys 433 (K433) enhances nuclear translocation of dimeric PKM2.^91^ This translocation enables PKM2-dependent phosphorylation of substrates such as STAT3 (Y705) and histone H3 (T11), thereby activating transcriptional programmes linked to proliferation and stress responses.^91^ Deacetylation by SIRT6 counteracts this process by promoting nuclear export and suppressing non-canonical PKM2 functions.^92^ Notably, emerging evidence indicates that p300 acetyltransferase activity is elevated in Alzheimer's disease, providing a potential mechanism by which disease-associated signalling may enhance PKM2 dimerization and nuclear function.^93,94^

### Ubiquitination and protein turnover

Ubiquitination and protein turnover provide an additional layer of control. Stress-responsive proteins, such as AK4, promote PKM2 degradation through Parkin—a RING-HECT hybrid E3 ubiquitin ligase—thereby enhancing mitochondrial function and energy metabolism in neurons.^95^ Parkin also monoubiquitinates PKM2 at specific lysine residues to reduce enzymatic activity without promoting degradation, shifting metabolism towards aerobic glycolysis in tumour cells.^96^

This balance is modulated by deubiquitinating enzymes (DUBs) including OTUB2^97^ and PSMD14,^98^ and by ubiquitin ligases such as TRIM35, which shifts PKM2 towards its active tetrameric form.^99^ Although TRIM35 does not alter total *PKM2* expression, it increases PKM2 enzymatic activity by reducing the dimeric fraction, thereby suppressing the Warburg effect and consequently limiting tumour growth, proliferation and epithelial–mesenchymal transition (EMT) while promoting apoptosis in breast cancer models.^99^ USP36, another DUB, also stabilizes the dimer form of PKM2 through deubiquitination, enhancing glycolysis and supporting breast cancer progression via the Warburg effect. This regulation is mediated by the miR-140-3p/USP36 axis, where miR-140-3p inhibits *USP36* expression, leading to increased PKM2 ubiquitination, degradation and consequent suppression of glycolysis and tumour growth.^100^

Overall, PKM2 is regulated through a tightly orchestrated network of PTMs, transcriptional and splicing mechanisms and non-coding RNA interactions. These regulatory layers control not only its glycolytic function but also its nuclear and signalling activities, which are increasingly recognized as contributing to Alzheimer's disease pathophysiology. While much of this knowledge comes from studies in cancer and proliferative cells, emerging evidence suggests that similar mechanisms may operate in neurons and glia under stress or in neurodegenerative disease, warranting further investigation.

## PKM2 dysregulation in Alzheimer's disease

Dysregulation of PKM2 contributes to Alzheimer's disease pathogenesis by linking metabolic reprogramming with key disease features. Deep DIA-MS proteomic analysis of CSF from 400 individuals, ranging from cognitively unimpaired to those with dementia, revealed a pronounced upregulation of glycolytic enzymes—including PKM, ALDOA, ENO1, LDHA and ALDOC—in Alzheimer's disease compared with non- Alzheimer's disease groups. Immunoblotting confirmed the presence of full-length PKM and ALDOA in CSF, and their levels negatively correlated with CSF glucose, reflecting altered glycolytic flux.^101^ Similar patterns were observed in brain tissue, with PKM expression elevated in regions affected by Alzheimer's disease pathology. Notably, CSF PKM levels were significantly higher in patients exhibiting both amyloid and tau pathology, regardless of disease stage. In preclinical Alzheimer's disease, individuals with elevated baseline CSF PKM experienced faster decline on memory recall tests, although global cognitive measures, such as the Mini-Mental State Exam, were not yet affected. This pattern was confirmed in an independent cohort, where CSF PKM was again elevated in tau-positive individuals.^102^ These findings suggest that PKM upregulation occurs early in disease progression and may serve as a sensitive biomarker of emerging cognitive impairment. Beyond its potential as a biomarker, PKM2 actively contributes to Alzheimer's disease pathology. It drives abnormal neuronal cell-cycle re-entry,^103–107^ enhances Aβ plaque formation^32,108^ and promotes Tau hyperphosphorylation^109,110^ through both transcriptional regulation and PTMs. In microglia, PKM2 overactivation sustains chronic neuroinflammation and impairs Aβ clearance.^31,111,112^ Collectively, these processes position PKM2 as a key mediator linking metabolic dysfunction to Alzheimer's disease pathobiology.

### The role of PKM2 dysregulation in cell cycle re-entry

Neuronal dedifferentiation refers to the process in which mature neurons lose their specialized identity and revert to a less differentiated state, often triggered by cell-cycle re-entry.^17^ Recent findings suggest that this phenomenon plays a central role in early onset familial Alzheimer's disease (EOFAD).^16^ During dedifferentiation, neurons adopt a mixed-lineage state characterized by altered chromatin accessibility, largely driven by the RE1-silencing transcription factor (REST). REST suppresses genes that maintain neuronal identity while activating precursor-like genes, effectively pushing neurons towards a less differentiated state. Transcriptomic studies of PSEN1-mutant patient brains reveal similar molecular signatures, further supporting the link between dedifferentiation and Alzheimer's disease pathobiology. This process is tightly regulated by chromatin remodelling, histone modifications and transcriptional regulators, ultimately causing neurons to acquire non-ectodermal lineage traits.^16^ Interestingly, dedifferentiation appears closely tied to aberrant cell-cycle re-entry in mature neurons.^113^

Traditionally, neurons were viewed as permanently arrested in the G0 phase, but growing evidence shows that they can re-enter the cell-cycle under certain physiological and pathological conditions. In neurodegenerative diseases—including Alzheimer's disease, Down syndrome, Huntington’s disease and Amyotrophic lateral sclerosis—this aberrant re-entry is strongly associated with neuronal dysfunction and death,^113,114^ and modelling studies suggest that once neurons re-enter the cycle under Alzheimer's disease -related stress, the process becomes irreversible.^115^

In this context, PKM2 becomes highly relevant: although PKM2 is best known for driving cell proliferation and cell-cycle entry in cancer, similar PKM2-dependent mechanisms appear to operate in Alzheimer's disease. Instead of supporting growth, however, PKM2-driven cell-cycle re-entry in neurons contributes to dedifferentiation and vulnerability, closely tied to the metabolic shift characteristic of the Warburg effect. PKM2’s central role in this rewiring positions it as a key factor connecting metabolic dysfunction to neuronal cell-cycle dysregulation and neurodegeneration.^103–107,116^ In Alzheimer's disease, the accumulation of pathological Tau is a key driver of neuronal cell-cycle re-entry, and thereby neurodegeneration. Since the early observation in 1996 linking Tau to p16 upregulation, research has shown that pathogenic Tau promotes cell-cycle re-entry through multiple mechanisms: overstabilization of the cytoskeleton,^117^ microtubule destabilization,^118^ nuclear structural changes,^119^ heterochromatin decondensation^120^ and activation of transposable elements.^17,121^ Emerging evidence further suggests that Tau may exert part of these effects through PKM2 signalling, positioning PKM2 as a potential mediator that connects Tau pathology to aberrant cell-cycle re-entry and downstream neurodegenerative processes.^103–107^

**Figure 3:**
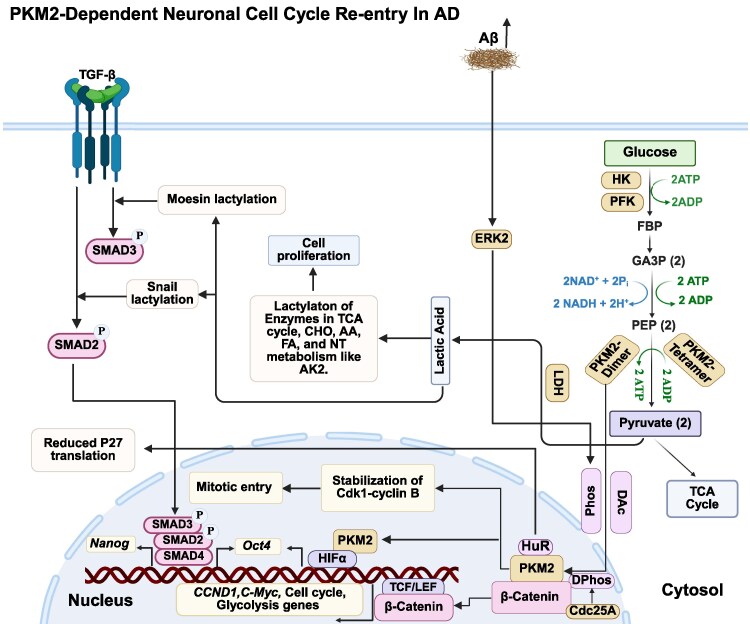
**PKM2 dysregulation induces cell-cycle re-entry.** PKM2 dysregulation induces neuronal cell-cycle re-entry, and thereby neurodegenerative processes. Through its interaction with β-catenin, PKM2 undergoes critical modifications, including dephosphorylation and deacetylation, enabling its nuclear translocation and interaction with β-catenin, which leads to the activation of genes involved in proliferation and metabolism. ERK2-mediated phosphorylation of PKM2 further enhances its nuclear function, while PKM2 reciprocally influences ERK activity, establishing a feedback loop relevant to neurodegeneration. Additionally, PKM2-driven lactate production contributes to cell-cycle re-entry, with lactylation modifying key proteins involved in cell-cycle progression. PKM2’s interaction with HuR regulates *p27* expression, impacting mitotic entry. As a coactivator of HIF-1α, PKM2 influences pluripotency-associated genes such as *Oct4*, which governs G1/S transition. It also stabilizes the Cdk1–Cyclin B complex, ensuring proper G2/M transition. In tauopathies, PKM2-induced lactate production facilitates Moesin lactylation, amplifying TGF-β/SMAD3 signalling and promoting neuronal cell-cycle re-entry. Furthermore, lactate modulates the TGF-β/SMAD2 pathway by inducing Snail1 lactylation, linking metabolic dysregulation to EMT and cell-cycle activation. These mechanisms collectively highlight PKM2’s role as a molecular bridge between metabolic shifts, neuroinflammation and aberrant cell-cycle reactivation in Alzheimer's disease. TGF-β, transforming growth factor beta; Aβ, amyloid beta; HK, hexokinase; PFK, phosphofructokinase; ATP, adenosine triphosphate; ADP, adenosine diphosphate; FBP, fructose-1,6-bisphosphate; GA3P, glyceraldehyde-3-phosphate; NAD, nicotinamide adenine dinucleotide (oxidized); NADH, nicotinamide adenine dinucleotide (reduced); SMAD, SMAD family of transcription factors; TCA, tricarboxylic acid cycle; CHO, carbohydrate; AA, amino acid; FA, fatty acid; NT, nucleotide; AK2, adenylate kinase 2; ERK2, extracellular signal-regulated kinase 2; PKM, pyruvate kinase M; PEP, phosphoenolpyruvate; Dac, deacetylation; Phos, phosphorylation; Cdc25A, cell division cycle 25A; TCF/LEF, T-cell factor/lymphoid enhancer-binding factor; HIF-α, hypoxia-inducible factor alpha; OCT4, octamer-binding transcription factor 4; Cdk1, cyclin-dependent kinase 1; HuR, human antigen R (ELAVL1); LDH, lactate dehydrogenase; CCND, cyclin D; C-Myc, cellular Myc oncogene.

Dysregulation of PKM2 can influence cell-cycle entry and progression through multiple mechanisms, which are summarized below (Fig. 3).

#### One potential mediator is β-catenin

Although PKM2-dependent β-catenin activation is well documented in cardiomyocytes and tumour cells, direct evidence for this mechanism in neurons is not yet available. However, the accumulation of PKM2 in the neuronal nucleus in Alzheimer's disease models—together with accompanying transcriptional reprogramming and metabolic remodelling in these normally post-mitotic cells—suggests that similar PKM2–β-catenin interactions may occur under pathological conditions.^116^

β-Catenin is a key effector of canonical Wnt signalling and regulates genes involved in proliferation, differentiation, survival and metabolism, functions that are impaired in Alzheimer's disease due to suppression of Wnt signalling in the diseased brain.^122–128^ In cancer and proliferative systems, PKM2 has been shown to modulate β-catenin activity through both PTMs and direct protein–protein interactions. For example, PKM2 dephosphorylation at Ser37 by CDC25A facilitates β-catenin translocation to the nucleus, where it activates transcription of c-Myc and other targets involved in glycolysis and cell-cycle progression, establishing a feedforward loop that supports the Warburg effect and G1/S transition.^129,130^ Additionally, deacetylation of PKM2 at Lys62 by HDAC8 enhances its nuclear localization and promotes its interaction with β-catenin, thereby increasing *CCND1* expression and promoting proliferation.^131–133^

Recent cancer studies further highlight a coordinated PKM2–β-catenin–c-Myc signalling module that shapes both cell-fate decisions and metabolic programmes. In head and neck cancers, PAK2 has been shown to activate c-Myc through PKM2, enhancing β-catenin signalling and promoting the Warburg effect and tumour growth.^134^ In breast cancer, this pathway also involves Survivin, an inhibitor of apoptosis, supporting proliferation and therapy resistance.^135^ Glioma studies show that pharmacological inhibition of PKM2–c-Myc (e.g. with trametinib) suppresses glycolysis and reduces tumour cell viability.^136^ Similarly, in ovarian granulosa cells, a PAK2/β-catenin/c-Myc/PKM2 axis regulates β-catenin nuclear localization and transcriptional output to prevent apoptosis.^137^

Importantly, c-Myc itself is increasingly implicated in Alzheimer's disease pathogenesis. While it promotes proliferation in cancer, aberrant c-Myc activation in post-mitotic neurons is associated with pathological cell-cycle re-entry and subsequent neurodegeneration. Vulnerable neurons in Alzheimer's disease exhibit signs of attempted cell-cycle re-entry, including elevated *c-Myc* expression; because mature neurons cannot divide, this ectopic activation ultimately leads to dysfunction and death.^138^ This pathological role of c-Myc has also been recapitulated *in vitro*: differentiated SH-SY5Y neurons, which normally show low basal MYC levels, rapidly upregulate *c-Myc* in response to neurotoxic stimuli such as camptothecin or Aβ, preceding activation of apoptotic markers like p53 and PARP cleavage, indicating that *c-Myc* upregulation is an early and potentially causal contributor to neuronal death.^139^ Phosphorylated c-Myc (c-Myc-P) has also been observed in dystrophic neurites and neurons containing NFTs in Alzheimer's disease and related tauopathies, suggesting a role in neuronal vulnerability.^140^ Consistent with this, c-Myc activation appears to occur early in the pathogenic cascade, preceding DNA damage responses, and its inhibition—such as with the anti-tumour antibiotic mithramycin—can confer neuroprotection.^139,141^ Notably, the regulation of c-Myc converges on pathways that are also dysregulated in Alzheimer's disease: both Ras/MAPK and PI3K/Akt/mTOR signalling, which control MYC activity in cancer, are aberrantly activated in Alzheimer's disease, suggesting shared upstream mechanisms that may contribute to pathological c-Myc activation in neurons.^142^ However, in contrast to this detrimental role under pathological conditions, c-Myc also plays a crucial physiological role in NSCs, where it helps maintain proliferation and mitochondrial health via the Wnt3a/β-catenin axis. Disruption of this pathway—example.g. by chronic low-dose cadmium exposure—leads to oxidative stress, mitochondrial dysfunction and impaired hippocampal function, effects that can be rescued by *MYC* overexpression.^143^

#### The connection between PKM2 and cell-cycle control extends further through extracellular signal-regulated kinase signalling

Extracellular signal-regulated kinase 2 (ERK2) can phosphorylate PKM2 at Ser37, promoting its nuclear translocation, where PKM2 functions as a coactivator of β-catenin and enhances transcription of genes such as c-Myc that drive metabolic reprogramming and proliferation.^144^ Conversely, PKM2 can modulate ERK activity: in a rat model of neuropathic pain, PKM2 inhibition reduced ERK activation and alleviated pain behaviours, demonstrating bidirectional regulation.^145^ This interaction is particularly relevant to neurodegeneration, as Aβ accumulation correlates with heightened ERK activity in Alzheimer's disease, which can trigger aberrant neuronal cell-cycle re-entry and death.^115^

#### PKM2-driven metabolic changes also contribute to cell-cycle dysregulation through lactate production and protein lactylation

As a key driver of the Warburg effect, PKM2 increases lactate levels, which can modify non-histone proteins and alter cell-cycle control. A recent lactylome study in human hepatocellular carcinoma (HCC) revealed widespread lysine lactylation on non-histone proteins, particularly metabolic enzymes. Specifically, lactylation of adenylate kinase 2 (AK2) at Lys28 reduces its kinase activity, impairing ADP ↔ ATP/AMP exchange. This metabolic disruption enhances HCC cell proliferation, invasion and metastasis, and higher levels of lactylated AK2 are associated with more aggressive tumours and poorer clinical outcomes.^146^

#### PKM2 may also influence cell-cycle entry through its interaction with the RNA-binding protein HuR

In the nucleus, PKM2 regulates HuR localization and suppresses translation of the cell-cycle inhibitor p27. When the PKM2–HuR interaction is disrupted, p27 levels increase, leading to defects in mitotic entry, centrosome amplification and reduced proliferation.^68^

As mentioned before, PKM2 may also function as a coactivator for HIF-1α, thereby regulating genes involved in pluripotency and potentially influencing cell-cycle progression, including OCT4.^147^ OCT4 is a key factor for maintaining pluripotency and plays a critical role in controlling the G1/S transition of the cell cycle.^148^ It achieves this by modulating the expression of several cell-cycle-related genes, such as downregulating *PP1* and upregulating *CDK4/6–Cyclin* D complexes. These changes lead to phosphorylation of the retinoblastoma protein, which facilitates progression into the S phase of the cell cycle.^149^ PKM2 can also interact with the Cdk1–Cyclin B complex, stabilizing it and promoting G2/M transition and mitotic entry.^150^

#### Cytoskeletal remodelling provides an additional layer connecting PKM2 to cell-cycle reactivation

Moesin, a cytoskeletal protein involved in cell shape and signal transduction, has emerged as a critical player in tauopathy.^151^ Transcriptomic profiling in post-mortem Alzheimer's disease brains and tau transgenic mice consistently identifies *Moesin* as a hub gene. *Drosophila* models show that Moesin activation drives filamentous actin accumulation, EMT-like changes and neuronal cell-cycle re-entry—even in the absence of pathogenic tau—with region-specific vulnerability.^151^ PKM2-derived lactate can lactylate Moesin at Lys72, enhancing TGF-β/SMAD3 signalling, which supports cell-cycle progression and EMT-like responses.^152,153^

In addition to TGF-β/SMAD3 signalling, the TGF-β/SMAD2 axis may also connect PKM2 dysregulation to aberrant cell-cycle re-entry. PKM2-driven glycolysis elevates lactate production, which is a key promoter of EMT.^154^ Mechanistically, lactate promotes EMT by inducing lactylation of the transcription factor Snail1, which enhances its nuclear translocation, upregulates *TGF-β* expression and activates the SMAD2 pathway. Reducing lactate production mitigates these EMT-promoting effects.^155^ The role of SMAD2 in cell-cycle promotion is further supported by its well-established functions in stem cell biology. In pluripotent stem cells, SMAD2 helps maintain the undifferentiated state by partnering with core regulators such as NANOG. As differentiation begins, however, SMAD2 shifts its binding preference towards transcription factors like EOMES, directing cells towards the endoderm lineage, one of the earliest embryonic fates.^156^

### The dual role of neuronal cell-cycle re-entry in neurodegeneration and survival

In Alzheimer's disease, inappropriate reactivation of the cell-cycle machinery in post-mitotic neurons initiates S-phase DNA replication without subsequent mitosis, leading to the formation of tetraploid or hyperploid neuronal populations that precede overt neurodegeneration. In human Alzheimer's disease brains, such neurons—particularly within the hippocampus and basal forebrain—can sustain nearly complete S-phase DNA synthesis for months before ultimately degenerating.^157,158^ Parallel findings in mouse models show that neuronal tetraploidization emerges early, preceding amyloid and tau pathology, and strongly correlates with cognitive impairment.^159,160^ These hyperploid neurons display oxidative stress, genomic instability and synaptic dysfunction, ultimately contributing to region-selective neuronal loss.^161^

Although hyperploid neurons can initially evade death, their compromised physiology makes them increasingly vulnerable. Unscheduled DNA synthesis and chromosomal imbalance drive morphological alterations, synaptic weakening and axon initial segment shortening defects that disrupt network activity and hasten cognitive decline. Indeed, hyperploidy accounts for nearly 90% of neuronal loss in the entorhinal cortex, highlighting the profound impact of abnormal DNA content on neuronal viability.^161,162^

Importantly, these genomic disturbances are amplified by a feedforward cycle of oxidative stress. Aberrant cell-cycle activity increases ROS production, which in turn exacerbates mitochondrial dysfunction and inflicts further DNA damage. Persistent double-strand breaks eventually overwhelm neuronal repair pathways, resulting in cell-cycle arrest, progressive genomic instability and death through apoptosis or pyroptosis.^163–167^

Within this context, PKM2-mediated lactylation emerges as a critical metabolic driver of neuronal vulnerability. Lactylation of Tufm at K286—one of the major mitophagy regulators—disrupts its interaction with Tomm40, impairs mitochondrial trafficking and suppresses mitophagy. This mitochondrial stagnation promotes apoptosis, and in models of traumatic brain injury, intensifies tissue damage. Notably, preventing Tufm lactylation (e.g. TufmK286R mutation) or applying mild hypothermia can restore mitophagy and reduce neuronal loss.^168^

PKM2 also contributes directly to DNA instability through its non-canonical nuclear kinase activity.^169^ In its dimeric form, PKM2 phosphorylates histone H2AX at Ser139 (γ-H2AX) using PEP rather than ATP—mechanistically linking elevated PKM2 to increased double-strand break signalling and chromosomal abnormalities. This process enhances E2F activity as part of the DNA damage response, which under sustained oxidative stress may shift from repair towards apoptosis.^115^ Adding further complexity, impaired DNA repair and aberrant cell-cycle activity can drive neurons into a senescence-like state defined by loss of mature neuronal functions and diminished genomic maintenance.^170^

Yet, cell-cycle re-entry is not uniformly deleterious.^171,172^ Work in *Drosophila* and mammalian systems suggests that under certain conditions, polyploid neurons can resist cell death.^173^ In mouse models of Aβ exposure, neurons that engage a controlled or transient cell-cycle programme exhibit greater resilience than those that do not.^174^ Similar observations in APP23 mice—including early geminin upregulation and its enrichment in human Alzheimer's disease tissue—suggest that early cell-cycle activation may constitute a broad neuronal stress response to Aβ toxicity.^175^ More recent studies of hippocampal neurons show that many neuronal cells can briefly enter an early cell-cycle state and then return to quiescence (G0/1) while preserving their identity. Under Aβ challenge, neurons that sustain this controlled re-entry show increased survival, whereas those with incomplete or absent activation remain more vulnerable. Thus, cell-cycle engagement can function as a transient protective mechanism—up to a point.^175^ Over prolonged periods, however, continued re-entry becomes maladaptive. Persistent activation risks driving dedifferentiation, genomic collapse and ultimately neurodegeneration.^113,176,177^ This duality underscores the complex role of cell-cycle re-entry in neurodegenerative diseases, such as Alzheimer's disease, where the balance between protective and pathological effects may determine the progression of the disease.

### Impaired PKM2 function due to aggregation in senescent cells

A major hallmark of ageing is the disruption of proteostasis, which is regulated by chaperone-mediated folding and degradation pathways like lysosomes and proteasomes.^178–180^ Among the proteins most sensitive to proteostatic collapse are tau, Aβ and α-synuclein—canonical drivers of neurodegenerative pathology. Recent evidence indicates that PKM2 also intersects with these ageing pathways. In senescent cells, PKM2 undergoes abnormal aggregation that reduces its catalytic activity and disrupts glycolytic flux—despite glycolysis typically being upregulated in senescence as a compensatory adaptation. This loss of PKM2 function accelerates metabolic dysfunction characteristic of the senescent state. Notably, PKM2 aggregates form in coordination with clusters of other glycolytic enzymes, indicating a broader reorganization of metabolic machinery during cellular ageing. Lysosomal pathways, particularly autophagy, play a key role in clearing these aggregates and preserving metabolic integrity. Importantly, small molecules such as K35 and K27 have been shown to dissolve PKM2 aggregates, restore glycolytic activity and reduce senescence-associated phenotypes.^181^

## PKM2-dependent lactylation in Alzheimer's disease beyond cell-cycle entry

**Figure 4:**
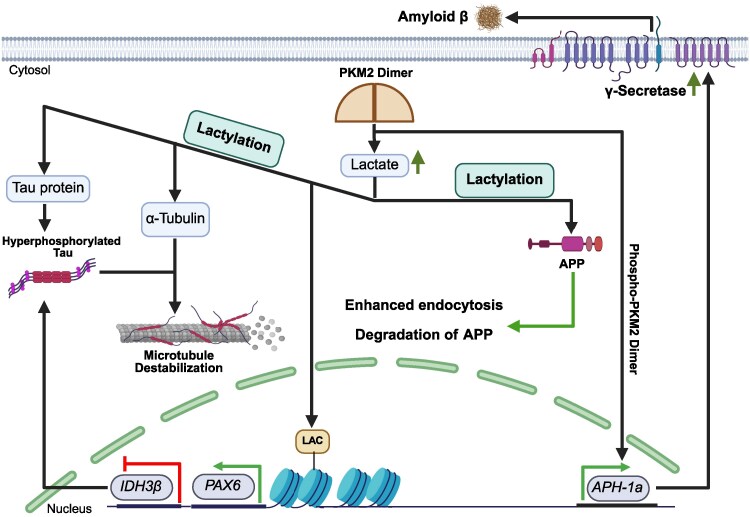
**PKM2-dependent lactylation in Alzheimer's disease beyond cell-cycle entry.** Dysregulated PKM2 dimerization enhances glycolysis, leading to lactate accumulation and subsequent protein lactylation. Lactylation modifies multiple targets in neurons: (i) α-tubulin, destabilizing microtubules and impairing intracellular transport; (ii) APP, reducing Aβ production via enhanced endocytosis and degradation; (iii) Tau protein, promoting hyperphosphorylation, aggregation and ferroptosis. Lactate also drives histone lactylation, which upregulates PAX6, inhibiting IDH3β and reinforcing Tau pathology. PKM2 additionally acts as a transcriptional coactivator, enhancing *APH-1a* expression and γ-secretase activity, increasing Aβ production. APP, amyloid precursor protein; LAC, lactate; APH-1a, anterior pharynx-defective 1a; PAX6, paired box 6; IDH3β, isocitrate dehydrogenase 3 beta.

PKM2-dependent lactylation introduces a layer of epigenetic and post-translational regulation that extends far beyond metabolism. Through its influence on key nuclear and cytoskeletal proteins, lactylation reshapes multiple cellular programmes relevant to Alzheimer's disease. These changes not only alter neuronal energy balance and promote maladaptive cell-cycle re-entry but also disrupt cytoskeletal organization, impair mitochondrial quality control and modulate Aβ production (Fig. 4).

### PKM2 disrupts cytoskeletal dynamics in neurodegeneration

Dysregulated PKM2 activity may contribute to neurodegenerative pathology in part by altering cytoskeletal stability. One emerging mechanism involves PKM2-dependent lactylation of α-tubulin—a recently identified PTM regulated by HDAC6. This modification occurs at Lys40 and, unlike α-tubulin acetylation (which stabilizes microtubules), lactylation increases microtubule flexibility in a reversible manner that depends on intracellular lactate levels.^182^ Under physiological conditions, this dynamic remodelling supports processes such as neurite outgrowth. However, when PKM2 activity becomes excessive, α-tubulin lactylation can become dysregulated, leading to microtubule destabilization. Such instability compromises intracellular transport and interacts with existing metabolic stresses, including mitochondrial dysfunction, to amplify axonal damage and neuronal vulnerability.^182^

### PKM2-mediated induction of Aβ production in Alzheimer's disease pathology

Emerging evidence indicates that PKM2 contributes to Alzheimer's disease progression by modulating amyloidogenic pathways, thereby offering an additional mechanistic link between PKM2 dysregulation and Alzheimer's disease pathology. Under hypoxic conditions—commonly observed in the Alzheimer's disease brain—*PKM2* expression is upregulated, and the protein shifts from its canonical glycolytic role to functioning as a transcriptional coactivator. In this context, phosphorylated PKM2 (Tyr105) dimerizes and enhances the transcription of *APH-1a*, a key component of the γ-secretase complex. This transcriptional activation increases γ-secretase activity, elevates Aβ production and exacerbates cognitive deficits in Alzheimer's disease models. Importantly, metabolic byproducts such as pyruvate and ATP do not directly influence γ-secretase activity, underscoring the pathological relevance of PKM2’s transcriptional functions under hypoxia. Consistent with these findings, transgenic Alzheimer's disease mice with elevated *PKM2* expression display greater Aβ accumulation and more severe memory impairments, whereas silencing *PKM2* ameliorates amyloid pathology.^32^

Although PKM2 activity is generally linked to detrimental effects in Alzheimer's disease —such as promoting amyloidogenic processing and increasing Aβ production—recent studies show that its impact can vary depending on the context and may even be protective under certain conditions. For example, lactylation of APP at Lys612 (APP-K612la) has emerged as a key modification influencing APP trafficking and metabolism. This modification reduces Aβ production and improves cognitive function in Alzheimer's disease models. The lactyl-mimetic mutant APP-K612T enhances APP endocytosis, prevents its interaction with BACE1 and facilitates its degradation via the endosomal–lysosomal pathway, collectively lowering Aβ accumulation.^183^ Importantly, this protective effects of lactylation depend on sufficient intracellular lactate, which serves as a driver of protein lactylation. Therefore, regulation of lactate production is critical for modulating amyloidogenic processing and maintaining neuronal health. In this context, VGLL4, a transcriptional cofactor involved in hypoxia sensing, enhances lactate synthesis by stabilizing LDHA, the enzyme responsible for lactate generation, whose activity is influenced by the dimeric form of PKM2. *VGLL4* expression is significantly reduced in the brains of Alzheimer's disease model mice and cells, whereas its overexpression increases LDHA levels and lactate production by preventing LDHA ubiquitination and degradation. This upregulation of lactate reduces amyloidogenic APP processing and mitigates synaptic damage, while inhibition of lactate production using sodium oxamate abolishes VGLL4’s protective effects, leading to elevated APP amyloidogenic processing.^108^

Together, these findings underscore the context-dependent nature of PKM2 and lactylation in amyloid pathology: while PKM2-mediated APP lactylation and VGLL4-driven lactate production can reduce amyloid pathology and support neuronal function, other lactylation events may promote Aβ accumulation.

### PKM2-mediated lactylation and its association with tau hyperphosphorylation

There is emerging evidence that disruption of metabolic homeostasis can drive pathological consequences relevant to tau in Alzheimer's disease. In brains of Alzheimer's disease patients and transgenic mouse models, IDH3β levels are markedly reduced, leading to impaired TCA-cycle activity, uncoupling of oxidative phosphorylation and accumulation of lactate.^109^ The increased lactate, in turn, promotes histone lactylation,^146^ which enhances expression of *PAX6,*^109^ a transcription factor ordinarily critical during CNS development.^184,185^ Once upregulated, PAX6 binds to the promoter region of *IDH3β* and suppresses its transcription, thereby exacerbating the original metabolic impairment—forming a ‘positive feedback loop’ of IDH3β-lactate-PAX6-IDH3β.^109^ Functionally, this feedback loop correlates with increased tau hyperphosphorylation, synaptic deficits and learning/memory impairment, as shown in cell models and Alzheimer's disease -transgenic mice.^109^

Moreover, separate evidence links PAX6 to the pathological phosphorylation of tau downstream of Aβ. In neuronal cultures treated with Aβ, PAX6 is induced via a cell-cycle signalling cascade (CDK/pRB → E2F1 → c-Myb → PAX6). Importantly, PAX6 directly transactivates GSK-3β, a kinase that phosphorylates tau at sites (e.g. Ser356, Ser396 and Ser404) linked to NFT formation. Knockdown of *PAX6* significantly reduces Aβ-induced *GSK-3β* upregulation and tau hyperphosphorylation and protects against neuronal death.^186^

Tau itself undergoes lactylation, which exacerbates Alzheimer's disease progression independently of phosphorylation. Elevated lactylation at K331 is associated with increased tau phosphorylation, cleavage and impaired ubiquitination, promoting tau aggregation and Alzheimer's disease pathology.^110^ Additionally, lactylation at K677 influences ferroptosis, an iron-dependent form of cell death. Reduced K677 lactylation suppresses microglial activation, protects neurons and improves cognition by modulating iron metabolism via the MAPK pathway. Inhibiting tau lactylation offers neuroprotection without directly altering tau phosphorylation.^187^

While metabolic dysregulation can influence tau phosphorylation, tau itself can reciprocally modulate neuronal metabolism. Specifically, tau drives metabolic reprogramming by upregulating PKM2 and promoting its association with membrane-bound complexes. In tau-expressing HEK293 cells and various mouse models (wild-type, tau knockout, human tau transgenic and 3xTg-AD), acute hyperglycaemic stress increases *PKM2* expression, shifting glucose metabolism towards aerobic glycolysis. Proteomic analyses further demonstrate that tau organizes membrane-associated metabolic complexes—including PKM2—by regulating their spatial localization, underscoring tau’s multifaceted role in coordinating neuronal energy metabolism.^29^

## PKM2 dysregulation in neuroinflammation: linking metabolic reprogramming to Alzheimer's disease progression

PKM2 serves as a central node linking cellular metabolism to inflammatory signalling, with its activity shaped by the balance between dimeric and tetrameric forms and by context-specific PTMs. Through interactions with regulators such as HIF-1α, SIRT5 and EGFR, PKM2 coordinates metabolic state with cytokine production and immune-cell activation.^188^ In the brain, elevated PKM2 levels are associated with heightened neuroinflammation and impaired waste-clearance pathways, illustrating how metabolic dysfunction can amplify inflammatory signalling.^189^ This link becomes particularly important given that brain-resident immune cells adopt distinct metabolic profiles depending on their activation state.

Macrophages exemplify this principle. Their metabolic configuration shifts according to their functional demands. Although traditionally framed within the M1/M2 dichotomy—in which M1 macrophages rely on aerobic glycolysis and M2 macrophages depend on oxidative phosphorylation,^190–193^ this binary classification is now recognized as overly simplistic. As Nahrendorf and Swirski^194^ emphasize, macrophage activation exists along a context-dependent spectrum shaped by tissue cues, developmental origins and disease stage. Nevertheless, metabolic reprogramming remains a critical component of macrophage function, and targeting glycolytic pathways continues to show promise as a strategy to modulate immune responses in inflammatory diseases.^195–197^ A similar metabolic adaptation occurs in microglial cells, the brain's resident immune cells. Upon exposure to inflammatory stimuli such as IFN-γ or Aβ, microglia shift towards a glycolytic phenotype, characterized by increased expression of key glycolytic enzymes, including *HKII* and *PKM2.*^143^ This shift is coupled to iron accumulation, which further impairs microglial chemotaxis and phagocytosis.^198,199^

PKM2 upregulation plays a central regulatory role in this process. While transient PKM2 activation supports glycolysis-driven phagocytosis,^31,111,112^ sustained activation disrupts both metabolic homeostasis and inflammatory control, promoting chronic neuroinflammatory signalling, synaptic loss and thereby cognitive decline. Pharmacological inhibition of PKM2 mitigates these outcomes by reducing pro-inflammatory cytokines and improving cognition.^111^

In Alzheimer's disease brains, microglia exhibit marked PKM2 upregulation, reflecting a shift towards glycolytic reprogramming. These PKM2⁺ microglia adopt a disease-associated, lipid-droplet-rich phenotype and preferentially localize near Aβ plaques, NFTs and blood vessels. However, their distribution suggests impaired chemotaxis, with many accumulating at a distance rather than clustering at plaque cores. Although PKM2⁺ microglia retain partial phagocytic capacity, they display phagocytic exhaustion (increased PLIN2), while PKM2⁻ microglia are largely non-phagocytic. This metabolic–functional decoupling contributes to reduced clearance of pathological aggregates and perpetuates chronic inflammation.^200^

**Figure 5:**
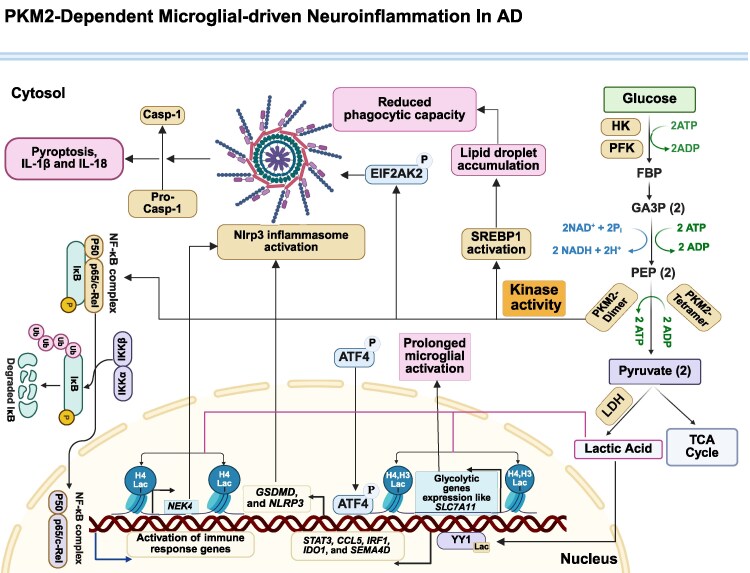
**This illustration depicts the intricate mechanisms by which PKM2 dysregulation contributes to neuroinflammation and Alzheimer's disease progression.** PKM2-driven lactylation in microglia sustains glycolysis and enhances histone lactylation, establishing a detrimental feedback loop that exacerbates neuroinflammation and impairs Aβ clearance. This cycle is further modulated by the transcription factor YY1, which upregulates inflammatory genes in microglia. PKM2 also mediates NLRP3 inflammasome activation, with elevated lactate levels inducing H4K12 lactylation and upregulating genes involved in NLRP3 inflammasome activation, thereby worsening Aβ accumulation. The activation of ATF2 and EIF2AK2 by PKM2 facilitates inflammasome activation, further contributing to neuroinflammation. Additionally, PKM2 influences neuroinflammation through SREBP1 activation, leading to metabolic dysfunction, mitochondrial damage, and a consequent reduction in the phagocytic capacity of microglial cells. The activation of NF-κB and excessive pro-inflammatory cytokine release in microglia further links PKM2 to neuroinflammation. Overall, PKM2 dysregulation drives a complex network of metabolic and inflammatory pathways that accelerate Alzheimer's disease progression. HK, hexokinase; PFK, phosphofructokinase; GA3P, glyceraldehyde-3-phosphate; NAD, nicotinamide adenine dinucleotide (oxidized); NADH, nicotinamide adenine dinucleotide (reduced); PKM, pyruvate kinase M; ADP, adenosine diphosphate; ATP, adenosine triphosphate; LDH, lactate dehydrogenase; TCA, tricarboxylic acid cycle; YY1, Yin yang 1 transcription factor; STAT3, signal transducer and activator of transcription 3; CCL5, C-C motif chemokine ligand 5; IRF1, interferon regulatory factor 1; IDO1, indoleamine 2,3-dioxygenase 1; SEMA4D, semaphorin 4D; NEK4, NIMA-related kinase 4; GSDMD, gasdermin D; NLRP3, NLR family pyrin domain containing 3; NF-κB, nuclear factor kappa B; H4lac, histone H4 lactylation; H3lac, histone H3 lysine lactylation; ATF4, activating transcription factor 4; IKK, IκB kinase; IκB, inhibitor of NF-κB; IL-1β, interleukin-1 beta; EIF2AK2, eukaryotic translation initiation factor 2-alpha kinase 2; SREBP1, sterol regulatory element-binding protein 1.

PKM2 contributes to microglia-driven neuroinflammation through multiple interconnected mechanisms (Fig. 5).

### PKM2-driven lactate production and lactylation

PKM2 promotes neuroinflammation partly by driving lactate-dependent epigenetic modifications. Elevated PKM2 increases lactate production, which enhances histone H4K12 lactylation—a modification that upregulates glycolytic genes, thereby reinforcing glycolytic flux and sustaining microglial activation.^30^ This creates a feedforward loop that intensifies inflammation, reduces Aβ clearance and accelerates Alzheimer's disease progression.^30,31,201^ Lactylation of YY1, a multifunctional transcription factor that regulates gene expression by acting as both an activator and repressor, further amplifies inflammatory signalling by increasing expression of *STAT3*, *CCL5*, *IRF1*, *IDO1* and *SEMA4D*; this process is regulated by p300, and inhibition of YY1 lactylation or p300 reduces microglial.^202^ Similar PKM2-lactylation pathways contribute to microglial activation in Parkinson’s disease, where P300/CBP-mediated H3K9 lactylation upregulates *SLC7A11* and drives neuroinflammation; glycolysis or SLC7A11 inhibition mitigates this response.^203^

Lactate's effects are context dependent. Chronic accumulation promotes inflammation, whereas transient elevations—such as during exercise—shift microglia towards a reparative phenotype and improve cognition in Alzheimer's disease -like models. Exogenous lactate reproduces these beneficial effects.^204^ PKM2 also supports adult hippocampal neurogenesis: its deficiency reduces lactate production and impairs hippocampal NSC/progenitor cell proliferation, while L-lactate supplementation restores neurogenesis and cognition via monocarboxylate transporter 2-mediated uptake.^205^

### PKM2-mediated activation of the NLRP3 inflammasome

PKM2 overactivation contributes to NLRP3 inflammasome signalling, thereby promoting IL-1β/IL-18 maturation and pyroptosis.^112,206–208^ In a rat model of hypoxic-ischaemic brain damage, elevated PKM2 levels in neurons correlated with increased inflammatory cytokines, including IL-1β and IL-18. Inhibiting PKM2 reduced the expression of key pyroptosis-related proteins, such as GSDMD-N, cleaved caspase-1 and NLRP3, while behavioural assessments indicated improvements in motor function, development and neurobehavioural outcomes in neonatal rats.^206^

ATF2 mediates part of this response. Lipopolysaccharide (LPS)-induced upregulation and phosphorylation of PKM2 activate ATF2, whereas PKM2 inhibition (with shRNA, 2-DG and TEPP-46) blocks ATF2 nuclear translocation and suppresses pyroptosis.^112^

The phosphorylation of EIF2AK2 is another key mediator linking metabolic reprogramming to inflammasome activation. Inhibiting the PKM2–EIF2AK2 pathway has been shown to suppress the activation of NLRP3 and AIM2 inflammasomes, leading to a reduction in the release of pro-inflammatory factors such as IL-1β, IL-18 and HMGB1. Notably, pharmacological inhibitors of PKM2, such as shikonin and C16, have demonstrated protective effects by effectively suppressing inflammasome activity.^209^ Lactate-mediated H4K12 lactylation also contributes to NLRP3 activation. In Alzheimer's disease models, exposure to cigarette smoke exacerbates Aβ plaque deposition and cognitive decline by increasing lactate levels. This rise in lactate triggers NLRP3 activation through H4K12 lactylation, which in turn impairs microglial autophagy, leading to further Aβ accumulation. Notably, treatments such as MCC950, a selective NLRP3 inhibitor, and oxamate, a competitive inhibitor of lactate LDH, have been shown to counteract these effects.^210^ H4K12 lactylation may promote NLRP3 activation by increasing *NEK7* expression, a crucial component of the inflammasome. Supporting this mechanism, inhibiting lactylation lowers NEK7 levels, reduces inflammasome activity and pyroptosis and improves cognitive function in Alzheimer's disease model mice.^211^

Evidence suggests that the relationship between PKM2 dysregulation and NLRP3 inflammasome activation is bidirectional. In this context, studies have shown that the NLRP3 inflammasome modulates glycolysis in macrophages through the IL-1β–PFKFB3 axis. Notably, treatment with MCC950 attenuated LPS + Aβ-induced glycolysis and suppressed *PFKFB3* expression without altering oxidative phosphorylation.^212^

### PKM2–SREBP1 axis and lipid dysregulation

SREBP1 also contributes to PKM2-driven neuroinflammatory signalling. In 3xTg-AD mice, SREBP1 activation induces lipid droplet accumulation, mitochondrial damage, impaired mitophagy and reduced phagocytic capacity. TRPV1 activation with capsaicin inhibits PKM2 dimerization, suppresses SREBP1 activity, restores microglial lipid homeostasis and alleviates neuroinflammation.^213^

### PKM2 and NF-κB-dependent inflammatory cascades

The activation of NF-κB provides another link between PKM2 dysregulation and neuroinflammation. In the PISE mouse model, microglial *PKM2* is significantly upregulated, contributing to excessive NF-κB activation and the release of pro-inflammatory cytokines (C1q, TNF-α and IL-1α). *PKM2* knockdown suppresses NF-κB signalling, reduces microglial activation, prevents astrocytic C3 upregulation and protects neurons via the C3–C3aR pathway.^214^

Collectively, these findings demonstrate that PKM2-driven metabolic reprogramming is a central determinant of microglial activation. In Alzheimer's disease and related neurodegenerative diseases, PKM2 promotes sustained glycolysis, pathological lactylation, inflammasome activation and lipid metabolic dysfunction. These changes impair microglial clearance of pathological aggregates and amplify chronic inflammatory signalling.

### The dual role of astrocytic PKM2 in neurodegeneration

Astrocytes display substantial heterogeneity in both morphology and function, which varies across developmental stages and disease states.^215^ This diversity is strongly shaped by underlying metabolic differences, influencing how astrocytes respond to CNS diseases pathology.^216^ In Alzheimer's disease, the role of astrocytic PKM2 remains contentious. While some studies suggest that activation of astrocytic PKM2 is linked to neuroinflammation in Alzheimer's disease,^217^ other evidence points to the neuroprotective role of astrocytic PKM2, particularly in providing lactate as an additional energy source for neurons.^218,219^

A clearer picture emerges when considering microglia–astrocyte crosstalk. Aβ-activated microglia stimulate the formation of A1-reactive astrocytes, which enhance glycolysis^220^ probably through the AKT–mTOR–HIF-1α–PKM2 pathway.^221^ This metabolic reprogramming enhances L-lactate production, which under neuroinflammatory conditions can impair synaptic plasticity by increasing oxidative stress and disrupting neuronal signalling.^221^ Notably, Aβ alone is insufficient to induce astrocytic glycolysis; rather, microglial activation amplifies this metabolic response. Consistent with this, mTOR inhibition with rapamycin reduces glycolysis-derived L-lactate and alleviates the associated neuronal dysfunction.^221^ Additional evidence shows that PKM2 nuclear translocation contributes to astrocyte activation and inflammation. In models of experimental autoimmune encephalomyelitis, nuclear PKM2 enhances glycolysis and inflammatory gene expression via *STAT3*, *NF-κB* and *c-Myc*. Blocking PKM2 translocation with small molecules such as DASA-58 or TEPP-46 suppresses astrocyte activation and mitigates disease severity.^217^ Accordingly, studies using D-galactose-induced memory impairment models show that inhibiting the activity of PKM2 with Shikonin restores redox balance, reduces astrocyte activation and pro-inflammatory cytokine production (TNF-α, IL-6 and IL-1β) and consequently improves learning and memory. Higher Shikonin doses produce stronger effects, indicating dose-dependent neuroprotection.^222^

Conversely, complete PKM2 loss in astrocytes can worsen neuronal outcomes. For example, PKM2 deletion impairs the astrocyte–neuron lactate shuttle, limiting neuronal access to energy substrates during metabolic stress such as global cerebral ischaemia. This results in energy depletion, oxidative damage and microtubule instability in neurons. Importantly, exogenous lactate can rescue these deficits.^219^

Together, these findings highlight that PKM2 exerts highly context-dependent effects in astrocytes—driving pro-inflammatory glycolysis in some settings while supporting neuronal energy metabolism in others—emphasizing that its activity must be precisely regulated and positioning PKM2 as both a potential therapeutic target and a sensitive marker of astrocyte metabolic state in Alzheimer's disease and other CNS diseases.

## Overview of PKM2 modulators in Alzheimer's disease treatment: research and outcomes

This section provides an integrated overview of natural and synthetic PKM2 modulators that have been explored for their potential relevance to Alzheimer's disease. Supplementary Table 2 summarizes each compound, the supporting experimental evidence and the associated biological outcomes, providing a framework for understanding how PKM2-directed interventions may influence pathogenic processes. These modulators act through diverse mechanisms—including transcriptional regulation, PTM and direct allosteric interaction—reflecting the multifaceted nature of PKM2 control. Examining these agents collectively clarifies how targeting PKM2 could mitigate key molecular pathways implicated in Alzheimer's disease.

Several natural compounds inhibit PKM2 via distinct mechanisms. ‘Shikonin’ inhibits PKM2 by directly reducing its enzymatic activity, blocking phosphorylation at Y105 and suppressing both its dimeric and tetrameric forms. It also prevents PKM2’s nuclear translocation, disrupting glycolysis.^223,224^ ‘Resveratrol’ inhibits PKM2 through multiple mechanisms, such as downregulating its mRNA and protein levels, which is associated with mTOR signalling inhibition. It may also regulate PKM2 via microRNA pathways.^225–228^ ‘Apigenin’ works as an allosteric inhibitor by binding to the K433 residue, suppressing PKM2 expression through the β-catenin/c-Myc/PTBP1 pathway.^229–231^ ‘Curcumin’ inhibits PKM2 by reducing its mRNA and protein levels, likely through suppression of the mTOR/HIF1α signalling axis. It acts as a non-competitive inhibitor and decreases PKM2 phosphorylation at Y105.^232–234^ ‘Melittin’ inhibits PKM2 by lowering its expression, suppressing the Warburg effect and reducing aerobic glycolysis.^235^ ‘Capsaicin’ inhibits PKM2 by binding directly to Cys424, reducing its expression, suppressing the Warburg effect and preventing PKM2 nuclear translocation and dimer formation.^213,236,237^ ‘Silibinin’ competitively inhibits PKM2 with high affinity (Ki = 0.61 µM) by binding directly to the PEP-binding site, resulting in potent enzymatic suppression (IC_50_ = 0.91 µM). In addition to inhibiting PKM2 activity, silibinin also reduces PKM2 expression levels.^238^ ‘Epigallocatechin-3-gallate’ inhibits PKM2 by reducing its expression, directly decreasing its activity. It may also alter PKM2’s oligomeric state and influence other metabolic pathways.^239–241^ ‘Kaempferol’ upregulates miR-326, which targets PKM2, blocks splicing factors promoting its expression and reduces PKM2 protein levels. It also modulates the miR-339-5p-PKM2 axis and suppresses aerobic glycolysis.^242,243^ ‘Quercetin’ inhibits PKM2 by reducing its expression through suppression of the Akt–mTOR pathway and promoting miR-326, which targets PKM2. It also inhibits PKM2’s nuclear translocation and reduces glycolysis.^244,245^ ‘Naringenin’ inhibits PKM2 by directly binding to the enzyme, interfering with its activity and reducing *PKM2* expression.^244,246,247^ ‘Berberine’ inhibits PKM2 by binding directly to it, disrupting its activity, promoting its ubiquitination and degradation, and modulating microRNA expression, such as upregulating *miR-145*, which further impacts PKM2 levels.^248–251^ ‘Genistein’ inhibits PKM2 by downregulating *HIF-1α*, which reduces *PKM2* expression and activity.^252^ ‘Caffeic acid’ inhibits PKM2 by downregulating its expression through the suppression of key signalling pathways.^244,253^ ‘Emodin’ inhibits PKM2 by enhancing the interaction between PKM2 and Nrf2, activating the Nrf2/ARE pathway. It also suppresses the mTOR and AKT pathways while activating AMPK, indirectly affecting PKM2 activity and expression.^254–256^ ‘Celastrol’ inhibits PKM2 by covalently binding to Cys31 and Cys424, which promotes PKM2 tetramer formation. It also downregulates signalling proteins such as Akt, HIF-1α and mTOR, further enhancing PKM2 suppression.^238,257,258^

Synthetic modulators also target PKM2 selectively. ‘TEPP-46’ activates PKM2 by acting as an allosteric activator, stabilizing its tetrameric form and enhancing PK activity.^259^ ‘Compound 3K (PKM2-IN-1)’ inhibits PKM2 by selectively binding to it, disrupting the PKM2 tetramer to promote monomer formation and inhibiting PK activity.^260^ ‘DASA-58’ activates PKM2 by binding allosterically to a distinct pocket on PKM2, stabilizing its tetrameric form and increasing its enzymatic activity.^259^

Collectively, these modulators demonstrate diverse mechanisms for fine-tuning PKM2 activity, offering potential therapeutic avenues for neurodegenerative diseases as well as cancer.

## Conclusion

Emerging evidence suggests a bidirectional relationship between PKM2 dysregulation and the core pathological features of Alzheimer's disease, namely Aβ accumulation, tau hyperphosphorylation and chronic neuroinflammation. Not only do these hallmarks processes alter *PKM2* expression and favour its less active dimeric and nuclear forms, but PKM2 dysregulation itself actively promotes and amplifies these disease mechanisms, creating a vicious cycle that accelerates neurodegeneration. A major downstream consequence of PKM2 dysregulation in neurons is aberrant cell-cycle re-entry and dedifferentiation, events increasingly recognized as central drivers of Alzheimer's disease pathology. These changes result from metabolic reprogramming through the Warburg effect, increased lactate production and activation of pathological signalling pathways, ultimately compromising neuronal identity and function. While transient cell-cycle re-entry may offer initial neuroprotective effects under stress, sustained activation is associated with the progression of Alzheimer's disease.

Modulating PKM2 is complex because its effects vary across cell types and states, reflecting its dual role in diseases. Understanding whether its pathological impact arises from a loss-of-function (glycolytic inefficiency) or gain-of-function (non-metabolic/nuclear activity) mechanism is essential for developing effective, targeted therapeutic strategies. In Alzheimer's disease and other neurodegenerative conditions, neurons show a shift towards the dimeric form of PKM2, which exhibits non-metabolic functions, including nuclear translocation, kinase activity and gene regulation. The dimeric represent both a loss-of-function in canonical glycolytic role and a gain-of-function in nuclear translocation and kinase activities.

Therapeutic approaches fall into two main categories: activators, which stabilize the tetramer to restore neuronal oxidative phosphorylation and prevent pathological nuclear and non-metabolic functions, exemplified by allosteric modulators TEPP-46 and DASA-58 or covalent binders such as celastrol; and inhibitors, which suppress the dimer’s non-canonical functions, including natural compounds (silibinin, shikonin and quercetin) and synthetic molecules (Compound 3K). Multi-target natural compounds, such as resveratrol and berberine, also show efficacy by modulating PKM2 and upstream pathways. The most promising modulators target the pathological dimeric form, such as Compound 3K and shikonin, or act on multiple pathways, as seen with resveratrol, curcumin and berberine, to mitigate Alzheimer's disease pathology.

PKM2’s context-dependent roles complicate therapy: in astrocytes, inhibition can reduce inflammatory activation but impair lactate shuttling to neurons, while in neurons, PKM2-driven cell-cycle re-entry and lactylation can be protective when transient but pathological when prolonged. These complexities underscore the need for cell- and context-specific modulation, potentially using advanced imaging and drug delivery technologies to selectively target activators to neurons and inhibitors to glia, supporting a nuanced therapeutic approach to mitigate PKM2-mediated pathology in Alzheimer's disease.

Recent advances have identified several new PKM2 modulators such as Dihydroartemisinin,^261^ Nevadensin and Asarinin, from *Zanthoxylum armatum,*^262^ ML-265,^263^ Cancerous inhibitor of protein phosphatase 2A,^258^ and Compound 3h,^264^ the efficacy of which in the context of Alzheimer's disease should be rigorously assessed. These modulators offer the potential to fine-tune PKM2 activity selectively, possibly restoring neuronal homeostasis and mitigating neurodegenerative processes.

Future research should also focus on elucidating PKM2’s mechanistic contributions to Alzheimer's disease pathology and evaluating the long-term safety and efficacy of emerging modulators in preclinical and clinical studies. Given that much of the current understanding derives from cancer biology, it is critical to determine which mechanisms are conserved or uniquely relevant in the Alzheimer's disease brain.

In parallel, investigating therapeutic strategies that simultaneously target multiple PKM2-related processes—such as metabolic reprogramming, aberrant cell-cycle activation and neuroinflammation—may offer additive or synergistic benefits. The use of integrated multi-omics analyses, advanced imaging technologies and improved drug-delivery platforms will be essential for defining the spatiotemporal dynamics of PKM2 activity in the Alzheimer's disease brain. Collectively, these approaches may accelerate the development of more precise and effective PKM2-based interventions for Alzheimer's disease and related neurodegenerative disorders.

## Supplementary Material

fcag054_Supplementary_Data

## Data Availability

Data sharing is not applicable to this article as no new data were created or analysed in this study.
