# Supplementary material for: Pyruvate kinase M2 in Alzheimer’s disease: from dysregulation to therapeutic inhibition
Source: Brain Commun. 2026 Feb 21;8(2):fcag054. doi: 10.1093/braincomms/fcag054 (PMC13012006; doi:10.1093/braincomms/fcag054)
Supplement: fcag054_Supplementary_Data [file fcag054_supplementary_data.docx]

| **Supplementary Table 1. List of Abbreviations and Their Definitions** | |
| --- | --- |
| **Abbreviation** | **Definition** |
| **2-DG** | **2-Deoxy-D-glucose**. |
| 3xTg-A | Triple transgenic mice |
| **AChE** | **Acetylcholinesterase** |
| **ACMSD** | **Amino Cyclopropane Carboxylate Deaminase** |
| AD | Alzheimer’s disease |
| **ADH3** | **Alcohol Dehydrogenase 3** |
| AK4 | Adenylate kinase 4 |
| AKT | Protein Kinase B |
| **ALDH2** | **Aldehyde Dehydrogenase 2** |
| ALS | Amyotrophic lateral sclerosis |
| **AMPAR** | **Alpha-amino-3-hydroxy-5-methyl-4-isoxazolepropionic acid receptor** |
| AMPK | AMP-activated protein kinase |
| APH-1a | Anterior Pharynx Defective 1a |
| APP | amyloid precursor protein |
| **ARE** | **Antioxidant Response Element** |
| **ATF2** | **Activating Transcription Factor 2** |
| ATM | Ataxia Telangiectasia Mutated |
| ATP | Adenosine Triphosphate |
| ATR | Ataxia Telangiectasia and Rad3-related |
| Aβ | Amyloid-beta |
| BACE1 | β-site APP Cleaving Enzyme 1 |
| **BDNF** | **Brain-Derived Neurotrophic Factor** |
| **C-Myb** | **Cellular Myelocytomatosis Viral Oncogene Homolog** |
| C-Myc | Cellular Myelocytomatosis Oncogene |
| **CaMKII-α** | **Calcium/Calmodulin-Dependent Protein Kinase II Alpha** |
| **CaMKKβ** | **Calmodulin-Dependent Protein Kinase Kinase Beta**. |
| Casp-1 | Caspase-1 |
| **CBP** | CREB Binding Protein |
| **CCL5** | C-C Motif Chemokine Ligand 5 (also known as RANTES) |
| CCND1 | Cyclin D1 |
| Cdc25A | Cell Division Cycle 25A. |
| CDK | Cyclin-Dependent Kinase |
| **CHOP** | **C/EBP homologous protein** |
| **COX-2** | **Cyclooxygenase-2** |
| **CREB** | **cAMP Response Element-Binding Protein** |
| **CSF** | **Cerebrospinal fluid** |
| **CT** | **Computed Tomography** |
| DAMPs | Damage-associated molecular patterns |
| DNA-PK | DNA-dependent Protein Kinase |
| E2F | E2 Transcription Factor |
| EGFR | Epidermal growth factor receptor |
| **EIF2AK2** | **Eukaryotic Translation Initiation Factor 2 Alpha Kinase 2** |
| **eIF2α** | **Eukaryotic initiation factor 2 alpha** |
| EOFAD | Early Onset Familial Alzheimer's Disease |
| ER | **Endoplasmic Reticulum** |
| ERK | Extracellular signal-regulated kinase |
| FBP | Fructose-1,6-bisphosphatase |
| FGFR1 | Fibroblast Growth Factor Receptor 1 |
| GA3P | Glyceraldehyde-3-phosphate |
| **GFAP** | **Glial Fibrillary Acidic Protein** |
| **GluA2** | **Glutamate receptor, ionotropic, AMPA type subunit 2** |
| **GluN2B** | **Glutamate receptor, ionotropic, NMDA 2B subunit** |
| **GLUT1** | **Glucose Transporter 1** |
| **GPX4** | **Glutathione Peroxidase 4** |
| **GRP** | **Glucose-Regulated Protein** |
| GSDMD | Gasdermin D |
| **GSH** | **Glutathione** |
| GSK-3β | Glycogen Synthase Kinase 3 Beta |
| HAT | Histone acetyltransferase |
| HDAC | Histone Deacetylase |
| HFD | High-fat diet |
| HIF-α | Hypoxia-Inducible Factor Alpha |
| HKII | Hexokinase II |
| **HMGB1** | **High Mobility Group Box 1** |
| hnRNPs | Heterogeneous ribonucleoproteins |
| **HSPB1** | **Heat Shock Protein Beta-1** |
| HuR | Human antigen R |
| IκB | Inhibitor of nuclear factor kappa B |
| **IDH3β** | **Isocitrate Dehydrogenase 3 Beta Subunit** |
| **IDO** | **Indoleamine 2,3-Dioxygenase** |
| **IFNγ** | **Interferon gamma** |
| IGF-1 | Insulin-Like Growth Factor 1 |
| IKK | IκB kinase |
| **IRF1** | Interferon Regulatory Factor 1 |
| JAK2 | Janus Kinase 2 |
| **JNK** | **C-Jun N-terminal kinase** |
| LDH | lactate dehydrogenase |
| LDH | Lactate dehydrogenase |
| **LED** | **Light Emitting Diode** |
| LncRNAs | Long non-coding RNAs |
| **LPS** | **Lipopolysaccharide** |
| **MAPK** | **Mitogen-Activated Protein Kinase** |
| **MCPIP1** | **Monocyte Chemotactic Protein-Induced Protein 1**. |
| **mCRP** | **Modified C-reactive protein**. |
| MEG3 | Maternally Expressed Gene 3 |
| **MMP9** | **Matrix metalloproteinase 9** |
| **MPTP** | **1-methyl-4-phenyl-1,2,3,6-tetrahydropyridine** |
| mTOR | Mechanistic Target of Rapamycin |
| **mtTFA** | **Mitochondrial Transcription Factor A** |
| NAD | Nicotinamide Adenine Dinucleotide |
| **NEK7** | **NIMA-related kinase 7** |
| **NEP** | **Neutral Endopeptidase**. |
| NF-κB | Nuclear Factor kappa-light-chain-enhancer of activated B cells |
| NFTs | Tau neurofibrillary tangles |
| **NMDAR** | **N-Methyl-D-Aspartate Receptor** |
| **NO** | **Nitric Oxide** |
| **NOD2** | **Nucleotide-binding Oligomerization Domain 2** |
| **NOS1** | **Nitric Oxide Synthase 1** |
| NPCs | Neural precursor cells |
| **NRF1/2** | **Nuclear Respiratory Factor 1 and 2** |
| **Nrf2** | **Nuclear factor erythroid 2-related factor 2** |
| NSCs | Neural stem cells |
| Oct4 | Octamer-binding transcription factor 4 |
| OTUB2 | OTU Deubiquitinase 2 |
| P-NF-κB | **Phospho-** NF-κB |
| **P300** | E1A Binding Protein P300 |
| PAMPs | Pathogen-associated molecular pattern |
| PAX6 | Paired Box 6 |
| PEP | Phosphoenolpyruvate |
| **PERK** | **Protein kinase RNA-like endoplasmic reticulum kinase** |
| PET | Positron Emission Tomography |
| PFK | Phosphofructokinase |
| PFKFB3 | 6-Phosphofructo-2-Kinase/Fructose-2,6-Bisphosphatase 3 |
| **PGC-1α** | **Peroxisome Proliferator-Activated Receptor Gamma Coactivator 1 Alpha** |
| **PGE2** | **Prostaglandin E2** |
| PHD3 | Prolyl Hydroxylase Domain-containing protein 3 |
| PI3K | Phosphoinositide 3-Kinase |
| PK | Pyruvate kinase |
| PKC | Protein Kinase C |
| PKL | Pyruvate kinase L |
| PKM2 | Pyruvate kinase M2 |
| PKR | Pyruvate kinase R |
| PP1 | protein phosphatase 1 |
| PPAR-γ | Peroxisome Proliferator-Activated Receptor Gamma |
| Pro-Casp-1 | Pro-caspase-1 |
| **PSD95** | **Postsynaptic Density Protein 95** |
| PSEN1 | Presenilin 1 |
| PSMD14 | Proteasome 26S Subunit, Non-ATPase 14 |
| PTBP1 | Polypyrimidine Tract Binding Protein 1 |
| PTEN | Phosphatase and Tensin Homolog |
| **RAGE** | **Receptor for Advanced Glycation End-products** |
| **Rb1** | **Retinoblastoma Protein 1** |
| **RIP2** | **Receptor Interacting Protein 2** |
| **RNS** | **Reactive Nitrogen Species** |
| ROS | Reactive oxygen species |
| RSV | Resveratrol |
| RSV-SeNPs | Resveratrol **-selenium nanoparticles** |
| **S100B** | **S100 calcium-binding protein B** |
| **SAMP** | **Senescence-Accelerated Mouse Prone**. |
| **SEMA4D** | Semaphorin 4D |
| **SIRT1** | **Sirtuin 1** |
| **SLC7A11** | **Solute Carrier Family 7 Member 11**. |
| **SOP** | superoxide dismutase |
| SREBP1 | Sterol regulatory element binding protein 1 |
| SRSF3 | Serine/Arginine-Rich Splicing Factor 3 |
| **STAT3** | Signal Transducer and Activator of Transcription 3 |
| STING | Stimulator of Interferon Genes |
| **STZ** | **Streptozotocin** |
| TCA | Tricarboxylic acid cycle |
| TCF/LEF | T-cell factor / Lymphoid enhancer-binding factor |
| **TFEB** | **Transcription Factor EB**. |
| TGF -β | Transforming Growth Factor Beta |
| **TLR4** | **Toll-Like Receptor 4** |
| **TNF-α** | **Tumor Necrosis Factor-alpha** |
| TOMM40 | Translocase of Outer Mitochondrial Membrane 40 kDa |
| TRIM35 | Tripartite Motif Containing 35 |
| **TrkB** | **Tropomyosin receptor kinase B** |
| **TRPV1** | **Transient Receptor Potential Vanilloid 1** |
| Tufm | Mitochondrial Translation Elongation Factor Tu |
| Ub | Ubiquitin |
| USP36 | Ubiquitin Specific Peptidase 36 |
| VGLL4 | Vestigial-Like Family Member 4 |
| YY1 | Yin Yang 1 |

| **Supplementary Table 2: PKM2 Modulators in AD: Research Overview and Outcomes** | | | | |
| --- | --- | --- | --- | --- |
| **Modulator** | **Evidence for PKM2 modulation** | **Experimental model** | **Sex of Experimental Animals/Humans** | **Experimental Outcome** |
| Shikonin (Natural naphthoquinone compound derived from the roots of *Lithospermum erythroidine*) | ^1,2^ | AlCl_3_-treated albino Wistar rats | M (Male) | Protecting against AlCl₃-induced Alzheimer’s by restoring behavioural changes and reducing oxidative stress, inflammation, and histological damage ^3^. |
|  |  | PC12 cells |  | Protecting against Aβ1–42-induced neurotoxicity by enhancing antioxidant defenses, stabilizing mitochondria, and inhibiting oxidative stress and apoptosis in PC12 cells ^4^. |
|  |  | C57BL/6 mice with alcoholic encephalopathy. | M | Inducing Neural Stem Cells and Neural Progenitor Cells proliferation, normalizes behavior in alcoholic encephalopathy mice, and mimics synthetic NF-ΚB and STAT3 inhibitors by enhancing neurogenesis ^5^. |
|  |  | MRL/MpJ-Faslpr (MRL/lpr) mice | F (Female) | Improving cognitive function, alleviated brain tissue damage, repaired blood-brain barrier permeability, and reduced neuroinflammation in NPSLE mice by inhibiting the β-catenin signaling pathway ^6^. |
|  |  | Primary microglial cells prepared from cerebral cortices of rat pups |  | Suppressing LPS-induced NO release and reduction in the production of several proinflammatory molecules from activated microglia ^7^. |
|  |  | BV2 microglial cells |  | Reducing microglial inflammation by inhibiting the NOD2/RIP2/NF-κB signaling pathway, offering protective effects ^8^. |
|  |  | BV2 microglial cells |  | Suppressing ROS generation and NF-κB signaling, thereby downregulating proinflammatory mediators, including NO, *PGE2*, and *TNF-α*, in LPS-stimulated BV2 microglial cells ^9^. |
|  |  | D-gal-induced Kunming mice | M | Acetylshikonin alleviates D-galactose-induced cognitive impairment and hippocampal aging in mice by reducing oxidative stress and neuroinflammation, upregulating *SIRT1*, and inhibiting the p53/p21 signaling pathway ^10^. |
|  |  | Sprague–Dawley rats | M | Promoting motor recovery and inhibiting of neuronal apoptosis by regulating ER stress through the PERK-eIF2α-CHOP signaling pathway and preventing Bax translocation ^11^. |
|  |  | SH-SY5Y cells and PC12 cells |  | Acetylshikonin and its derivatives, as novel acetylcholinesterase inhibitors, exert potent neuroprotective and antiapoptotic effects by upregulating heme oxygenase 1 and inhibiting oxidative stress ^12^. |
|  |  | SH-SY5Y cells |  | Inhibiting tau aggregation, including oligomerization, fibrillization, and droplet formation, and protecting neuroblastoma cells from tau-induced toxicity ^13^. |
|  |  | BV2 microglial cells– HT-22 murine hippocampal neuronal cells– HL-60 human monocytic cells |  | Modulating microglial immune functions independently of NLRP3 inflammasomes, reducing neurotoxicity and inflammation while enhancing phagocytosis ^14^. |
|  |  | Sprague-Dawley rats with hypoxic-ischemic brain damage | M&F | Reducing hippocampal neuronal pyroptosis through mitigating NLRP3/Caspase-1/GSDMD pathway and alleviating neuronal damage and inflammation ^15^. |
| Resveratrol (a naturally occurring polyphenolic compound found in various plants, particularly in grapes, berries, and red wine) | ^16-19^ | 30 selected patients with moderate to mild AD | M&F | Potential neuroprotective effects in moderate to mild AD by reducing Aβ accumulation and toxicity in the brain, which helps alleviate neuroinflammation ^20^. |
|  |  | 3xTg-AD | M | Neuroprotective effects in an AD mouse model by reducing Aβ toxicity, neuroinflammation, apoptosis, and autophagy ^21^. |
|  |  | Randomized, double-blind, placebo-controlled- Thirty-nine subjects with mild to moderate AD | M&F | Low-dose resveratrol is safe and well tolerated, showing trends of less cognitive and functional decline compared to controls, though not statistically significant ^22^. |
|  |  | PC12 cell line |  | Protection against Aβ1–42-induced neuronal damage in an AD cellular model by enhancing mitophagy, reducing apoptosis, and decreasing oxidative stress ^23^. |
|  |  | Tg19959 mice | M&F | Reducing Aβ plaque formation in a region-specific manner in mice, possibly by altering brain glutathione and cysteine levels ^24^. |
|  |  | SAMP8 and SAMR1 mice | M | Long-term dietary resveratrol increases life expectancy, enhances neuroprotection, reduces amyloid burden, and mitigates tau hyperphosphorylation in an age-related AD mouse model ^25^. |
|  |  | CSF and plasma samples from a subset of AD patients with baseline CSF Aβ42 <600 ng/ml | M&F | Reducing CSF MMP9, modulated neuroinflammation, and improved cognitive and functional outcomes in AD, potentially by enhancing adaptive immunity and maintaining blood-brain barrier integrity ^26^. |
|  |  | HEK293 cells stably transfected with human APP_695_ |  | Reducing Aβ levels in AD models by promoting its intracellular degradation through the proteasome ^27^. |
|  |  | APPswe/PS1dE9 mice | M | Long-term resveratrol treatment in the AβPP/PS1 mouse model of AD prevents memory loss, reduced amyloid burden, and increased mitochondrial function, primarily through the activation of SIRT1 and AMPK pathways ^28^. |
|  |  | HFD + AlCl_3_ + D-gal-treated ICR mice | M | Improving cognition in AD mice with metabolic disorders by balancing gut microbiota, reducing inflammation, oxidative stress, and insulin resistance, and inhibiting Aβ and Tau aggregation ^29^. |
|  |  | AlCl3-treated-Wistar rats | M | RSV-SeNPs enhance neuroprotection in an AD rat model by reducing oxidative stress, neuroinflammation, and cholinergic deficits while promoting Aβ clearance, inhibiting Tau phosphorylation, and modulating Sirt1/miRNA-134/GSK3β pathway ^30^. |
|  |  | SH-SY5Y cell line |  | Reducing Aβ1–42 aggregation in AD by cleaving it into smaller, non-aggregating fragments, inhibiting β-secretase activity, and reducing APP expression ^31^. |
|  |  | Colchicine-treated albino Wistar rats | M | RSV and its combination with donepezil reduces oxidative stress in a colchicine-induced Alzheimer’s rat model by lowering malondialdehyde levels and increasing SOD activity ^32^. |
|  |  | D-gal+AlCl3-treated-ICR mice–PC12 cells and BV2 cells | M | Resveratrol-selenium-peptide nanocomposite (TGN-Res@SeNPs) enhances cognitive function in an Alzheimer’s disease mouse model by crossing the blood-brain barrier, reducing Aβ aggregation, mitigating oxidative stress and neuroinflammation ^33^. |
|  |  | BV2 microglial cells |  | Protection against mCRP-induced neuroinflammation in microglia by modulating SIRT1, Nrf2, and NF-ĸB pathways, reducing oxidative stress and proinflammatory responses linked to Alzheimer’s risk ^34^. |
|  |  | D-gal+AlCl3-treated Swiss mice (SWR/J) | M | Co-treatment with Melatonin and RSV provided additive effects on cognitive performance, likely through the modulation of cholinergic transmission and BDNF/CREB signaling pathways in the prefrontal cortex ^35^. |
|  |  | STZ and subsequent Aβ1–40 hippocampal injected-Wistar rats | NM | Prevention of neurodegeneration in a rat model of diabetes and AD by activating Sirt1 signaling, which regulates the cholinergic system, reduces oxidative stress, and controls inflammation ^36^. |
|  |  | Scopolamine injected Sprague-Dawley rats | M | Neuroprotective effects in rats with scopolamine-induced AD by reducing oxidative stress, inflammation, and acetylcholinesterase levels, with vitamin E enhancing resveratrol's efficacy in managing cognitive impairment ^37^. |
|  |  | 5XFAD mice | M | Dihydro-resveratrol improves cognitive function and reduces neuroinflammation, amyloid precursor protein pathology, and NLRP3 inflammasome activation in AD mice by enhancing autophagy and mitophagy ^38^. |
|  |  | STZ-induced  Sprague Dawley rats | M | Oxyresveratrol-β-cyclodextrin improves cognitive function and reduces oxidative stress and HDAC activity in an AD rat model ^39^. |
|  |  | *Drosophila melanogaster* | M | Improving memory deficits and reducing Aβ aggregation in a Drosophila AD model, with its effects mediated partly through the activation of Sir2 ^40^. |
|  |  | Aged C57Bl/6 mice | M&F | Prolonged administration of resveratrol and copper (R–Cu) in mice reduces biological hallmarks of aging, including DNA damage, inflammation, and amyloid deposition ^41^. |
|  |  | Tg19959 Mice–Human brain samples–Primary cortical neurons dissected from E18 Sprague Dawley rats | M&F | Cis-resveratrol enhances tyrosyl-tRNA synthetase (TyrRS) activity and DNA repair, offering neuroprotection, while increased serum tyrosine levels during aging and neurodegenerative disorders can deplete TyrRS and cause neuronal DNA damage ^42^. |
| Apigenin (a flavonoid found in various plants, such as parsley, chamomile, and celery) | ^43-45^ |  |  | Apogenin binds to the hydrophobic cavity of the Aβ42 protofibril, disrupting its structure by targeting the anti-parallel β-strand. It destabilizes the fibril through electrostatic interactions and hydrogen bonds, potentially entering its interior due to its smaller size ^46^. |
|  |  | C57BL/6 strain mice | M | Neuroprotective effects in Parkinson’s disease by modulating pro-inflammatory and anti-inflammatory cytokines ^47^. |
|  |  | GFAP-IL6 heterozygous mice | M&F | Reducing microglia activation in the hippocampus of GFAP-IL6 mice, a model for age-related neurodegenerative disease, but not improving spatial memory recall, despite reducing neuroinflammation ^48^. |
|  |  | Swiss albino mice | M | Protection against LPS-induced neurotoxicity in mice by preserving the NAD+/NADH ratio, boosting mitochondrial SIRT3 activity, promoting mitochondrial biogenesis, and enhancing mitophagy to maintain mitochondrial function ^49^. |
|  |  | Scopolamine-induced mouse model of AD | M | Improving cognitive and memory function by regulating apoptosis, amyloidogenesis, and BDNF/TrkB signaling pathways, while also reducing lipid peroxidation and promoting Aβ degradation ^50^. |
|  |  | Aβ folding reporter SH-SY5Y cells |  | Activating the TRKB receptor, reducing Aβ aggregation, oxidative stress, and promoting neuroprotection in an in vitro model ^51^- |
|  |  | Wistar rats injected with Aβ | M | Protecting against Aβ–induced neurotoxicity in an AD rat model by inhibiting GSK-3β, which lowers tau hyperphosphorylation and suppresses *BACE1* expression, while not affecting GSK-3α levels ^52^. |
|  |  | Glial and neuronal cells obtained from the brain hemispheres of Wistar rats |  | Showing neuroprotective and anti-inflammatory effects in in vitro AD models by preserving neuronal and integrity, reducing microglial activation and inflammatory cytokine expression, and enhancing BDNF levels ^53^. |
|  |  | SIM-A9 microglial cells |  | Inhibiting IL-31 and IL-33 production by suppressing ERK, JNK, NF-κB, and STAT3 signaling pathways—thereby preventing nuclear translocation and DNA binding ^54^ |
|  |  | SH-SY5Y-derived ΔK280 Tau_RD_-DsRed cells |  | Reducing tau aggregation, oxidative stress, and caspase-1 activity while enhancing neurite outgrowth by restoring HSPB1 and NRF2 levels and activating TRKB-mediated ERK/CREB signaling ^55^. |
|  |  | Mouse microglial MG6 cells |  | Reversing the dysregulation of tryptophan metabolic enzymes—upregulating ACMSD and downregulating IDO—while suppressing IL-6 and NO production via inhibition of Erk, JNK MAPK, and NF-κB signaling ^56^ |
|  |  | C57BL/6 strain mice injected with MPTP | M | Mitigating MPTP-induced histopathological changes and reversed the dysregulation of cytokines, protecting motor neurons from inflammatory damage and demonstrating both prophylactic and therapeutic potential in a Parkinson’s disease mouse model ^47^. |
|  |  | Ovariectomized Wistar rats | F | Reducing hippocampal Aβ plaque formation, improved memory and learning deficits, and decreased caspase-3 expression ^57^, |
|  |  |  |  | Significant cholinesterase inhibition and antioxidant properties ^58^ |
|  |  | Transgenic *Drosophila melanogaster* |  | Alleviating AD symptoms in a transgenic Drosophila model by reducing oxidative stress, delaying motor impairment, inhibiting acetylcholinesterase activity, and preventing Aβ-42 aggregation in a dose-dependent manner ^59^. |
|  |  | SH-SY5Y cells |  | Protection from oxidative stress by scavenging ROS and RNS, enhancing cell viability, reducing apoptosis-related protein expression, and upregulating anti-apoptotic markers ^60^. |
| Curcumin (The active compound in turmeric) | ^61-63^ | APP/PS-1 transgenic mice | M | MAN-modified curcumin and ginsenoside Rb1 liposomes effectively cross the blood-brain barrier via GLUT1 transport, reducing oxidative stress, neuroinflammation, and apoptosis in APP/PS-1 mice ^64^ |
|  |  | BV2 cells and primary microglia—Mouse model with intra cerebroventricular LPS injection into the lateral septal complex region. |  | Promoting microglial M2 polarization via the CaMKKβ-AMPK pathway, reducing neuroinflammation ^65^ |
|  |  | APPsw Tg2576 transgenic mice–SH-SY5Y cells |  | Crossing the blood-brain barrier, binds amyloid, and inhibits Aβ aggregation, fibril, and oligomer formation, reducing amyloid levels in aged Tg2576 mice ^66^ |
|  |  | *Culex pipiens larva* |  | Inhibiting acetylcholine esterase in *Culex pipiens ^67^* |
|  |  | Primary astroglial cell cultures |  | Reducing inflammation in Aβ-treated astrocytes by acting as a PPARγ agonist, reversing COX-2 upregulation and NF-κB activation ^68^. |
|  |  | A Triple-Blind, Placebo-Controlled Clinical Trial–124 participants | F | Improving cognitive function, including memory, inhibitory control, and attention, in women with PMS and dysmenorrhea ^69^ |
|  |  | 5XFAD transgenic AD model |  | The quinoline-derived half-curcumin-dioxaborine probe effectively detects Aβ oligomers, offering a potential diagnostic tool for early AD detection by analyzing cerebrospinal fluid and differentiating stages of AD progression ^70^. |
|  |  | 5XFAD transgenic mice |  | Long-term, low-dose dietary curcumin intake in mice with AD improves short-term memory, reduces Aβ deposition and tau phosphorylation, delays disease onset, and extends lifespan ^71^. |
|  |  | C57BL/6J mice induced with normobaric hypoxia | M | Improving cognitive deficits in hypoxic-brain injury mice by promoting neurogenesis, enhancing dendritic spine growth, and increasing the expression of *BDNF* and *PSD95*, thereby supporting neuroplasticity and cognitive function ^72^. |
|  |  |  |  | This study identified 49 targets of curcumin for treating AD, highlighting key pathways and genes such as *GSK3B* and *NFKB1*, and suggesting that curcumin could improve AD pathology through multitarget and multipathway regulation ^73^ |
|  |  | SH-SY5Y cells– *Caenorhabditis elegans* |  | A dual modulator combining a curcumin-based GSK-3β inhibitor and a diethyl fumarate Nrf2 inducer effectively inhibits GSK-3β, activates Nrf2, and provides neuroprotective effects against Parkinson’s disease by enhancing neuronal resistance to oxidative stress and protecting against neurotoxicity ^74^. |
|  |  |  |  | Curcumin effectively reduces nanoscopic Aβ aggregates in synthetic brain membranes by utilizing membrane-mediated pathways, offering an alternative to peptide-targeting drugs for inhibiting amyloid aggregation ^75^. |
|  |  | B6C3-Tg (APPswe, PSEN1dE9) 85Dbo/NJU transgenic mice (also known as APP/PS1 transgenic mice) | M | Chronic oral administration of curcumin in an AD mouse model improves cognitive function and reduces neuroinflammation by inhibiting the HMGB1-RAGE/TLR4-NF-κB signaling pathway, without affecting amyloid plaque formation ^76^ ^76^. |
|  |  | BV2 microglial cells |  | The curcumin prodrug CUR-2GE, designed for improved stability and enhanced lipophilicity, effectively reduces neuroinflammation in LPS-stimulated microglial cells ^77^. |
|  |  |  |  | Bis-iodine-labeled curcumin demonstrated strong binding affinity for Aβ1-40 and Aβ1-42 aggregates, effective brain uptake, low toxicity, high stability, and potential as a targeted CT imaging agent for detecting Aβ plaques ^78^ |
|  |  | SH-SY5Y cell line |  | Curcumin effectively reduced oxidative stress, decreased ROS generation, inhibited tau hyperphosphorylation, and protected SH-SY5Y cells from Aβ oligomers-induced damage ^79^. |
|  |  |  |  | Curcumin, through keto-enol tautomerism, disassembles pentameric Aβ42 oligomers ^80^. |
|  |  | Primary astrocyte cultures from Wistar rats |  | Curcumin and piperine modulate astrocytic functions, including glutamatergic metabolism and the secretion of inflammatory markers like TNF-α and S100B ^59^ |
|  |  | SH-SY5Y cell line–5XFAD mouse model |  | The use of a photolabile curcumin analogue, CRANAD-147, combined with LED or molecular light reduces Aβ accumulation and neurotoxicity in AD ^81^. |
|  |  |  |  | Curcumin inhibits Aβ fibrillar oligomer (FO) aggregation on anionic membranes by embedding into the membrane, causing structural changes and reducing FO binding, likely through both membrane alterations and direct interactions with the FO surface ^82^. |
|  |  | C57BL/6 J mice injected with Aβ1–42 peptides | M | Curcumin improves cognitive function and alleviates brain tissue damage in AD mice by reducing inflammation, oxidative stress, and activating the AMPK pathway ^83^ |
|  |  | Sprague Dawley rats injected with STZ | M | Curcumin exhibits therapeutic and protective effects in STZ-induced diabetic rats by reducing Aβ and tau protein accumulation, lowering oxidative stress, and enhancing antioxidant activity ^84^ |
|  |  | Wistar rats injected with STZ | M | Nanocurcumin, but not natural curcumin, prevents STZ-induced memory impairment in rats by reducing hippocampal apoptosis and restoring Akt and CaMKII-α signaling ^85^ |
| Melittin (a principal constituent of honeybee venom) | ^86-88^ | C57BL/6 induced with D-gal and LPS | M | Melittin protects against cerebral ischemia by reducing inflammation and enhancing neuroprotection, with MCPIP1 playing a key role in its anti-inflammatory effects ^89^. |
| Capsaicin (The active compound found in chili peppers) | ^90-92^ | 3xTg transgenic mice–BV2 cells | M&F | Capsaicin activates TRPV1 to inhibit PKM2-SREBP1, reducing microglial lipid accumulation, neuroinflammation, and cognitive decline in AD ^90^. |
|  |  | 3xTg transgenic mice–BV2 cells | M&F | Capsaicin activates TRPV1 to induce autophagy, enhance microglial metabolism, and promote Aβ and phosphorylated tau clearance, improving cognitive function ^93^. |
|  |  | Sprague-Dawley rats injected with STZ | M | Dietary capsaicin reduced AD-associated tau hyperphosphorylation in T2D rats by activating PI3K/AKT and inhibiting GSK-3β ^94^. |
|  |  | A total of 338 participants aged 40 years or older |  | A capsaicin-rich diet was positively associated with cognitive function (Mini-Mental State Examination scores) and inversely correlated with serum Aβ40 and total Aβ levels ^95^. |
|  |  | C57Bl/6 mice microinjected with Aβ42 |  | Capsaicin-mediated TRPV1 activation improved spatial memory, synaptic function, and hippocampal LTP while reducing synapse loss in Aβ42-induced AD mice ^96^ |
|  |  | SH-SY5Y cell line |  | Capsaicinoids exhibit both beneficial and detrimental effects in AD, reducing Aβ aggregation and tau hyperphosphorylation while also increasing amyloidogenic APP processing and Aβ levels ^97^ |
|  |  | APP23/PS45 transgenic AD |  | Capsaicin improves synaptic plasticity, spatial learning, and memory by activating TRPV1, inhibiting GluA2-dependent AMPAR endocytosis, reducing Aβ deposition, and alleviating AD-related neuropathologies ^98^. |
|  |  | Sprague-Dawley rats administered with Okadaic Acid | M | Capsaicin reduces neurodegeneration and restores spatial memory in an AD-like experimental model by correcting altered levels of TNF-α, IL-1β, caspase-3, Aβ, p-Tau, and p-GSK-3β in the cortex and hippocampus ^99^. |
|  |  | APP/PS1 transgenic (Tg) mice–SH-SY5Y-APP695 cells | M&F | Capsaicin reduces brain Aβ burden, alleviates tau hyperphosphorylation, and improves cognitive decline by promoting non-amyloidogenic processing of APP ^100^ |
| Silibinin (A flavonoid derived from milk thistle) | ^101-104^ | APP/PS1 double Tg mice– Sprague-Dawley rats – *Drosophila melanogaster* |  | Silibinin inhibits acetylcholinesterase activity, reducing Aβ aggregation, improving cognitive function in transgenic mice, and promoting neurogenesis ^105^. |
|  |  | Sprague–Dawley rats injected with STZ –HT22 cells | M | Silibinin inhibits over-activation of GluN2B-containing NMDARs, which prevents calcium overload, reduces tau phosphorylation, and Aβ deposition, while promoting the CaMKIIα/BDNF/TrkB signaling pathway to enhance synaptic function and improve cognitive outcomes ^106^. |
|  |  | APP/PS1mice | M | Silibinin and silymarin improved memory and reduced amyloid plaques in APP/PS1 mice, regulating gut microbiota ^107^. |
|  |  | C57BL/6 mice injected with Aβ42– SH-SY5Y cells | M | Silibinin-loaded macrophage-derived exosomes effectively inhibited Aβ aggregation, reduced astrocyte activation, and alleviated cognitive impairment in AD mice by regulating the NF-κB pathway ^108^. |
|  |  | SH-SY5Y cell line |  | Silibinin encapsulated in human serum albumin nanoparticles enhanced its antioxidant and neuroprotective effects, improving cell viability and reducing oxidative stress and apoptosis in LPS-induced neuron-like cells ^109^ |
|  |  | Sprague–Dawley rats injected with STZ | M | Silibinin protects against ferroptotic damage and neuroinflammation in a sporadic AD model by inhibiting the p53/SLC7A11/GSH/GPX4 axis and blocking the STING-dependent inflammatory pathway, improving cognitive function and reducing anxiety/depression-like behaviors ^110^. |
|  |  | Sprague–Dawley rats injected with STZ | M | Silibinin protects against memory loss, neuronal damage, and tau hyperphosphorylation in an STZ-induced Alzheimer's model, while restoring insulin signaling ^111^ |
|  |  | APP/PS1 mice | M | Silibinin improves cognitive function in APP/PS1 mice by reducing Aβ deposition, downregulating *APP* and *BACE1*, upregulating *NEP*, and exerting antioxidant effects ^112^. |
|  |  | C57BL/6 injected with MPTP | M | Silibinin alleviates cognitive dysfunction in a MPTP-induced Parkinson’s disease mouse model by reducing α-synuclein aggregation, enhancing mitochondrial dynamics, decreasing oxidative stress, and protecting hippocampal neurons from apoptosis ^113^. |
|  |  | APP/PS1 mice | M | Silibinin improves memory impairments in APP/PS1 transgenic mice by reducing oxidative stress, inhibiting apoptosis through the JNK/Bax/caspase-3 signaling pathway, and enhancing synaptic protein levels ^114^. |
|  |  | C57 mice injected with formaldehyde | M&F | Silibinin improves cognitive dysfunction in formaldehyde-induced mice by activating the Nrf2 pathway to enhance antioxidant capacity, increasing the expression of formaldehyde-degrading enzymes *ADH3* and *ALDH2*, and inhibiting GSK-3β phosphorylation to reduce tau hyperphosphorylation^115^ |
|  |  | Sprague-Dawley rats injected with Aβ1-42 | M | Silibinin alleviates Aβ-induced anxiety and depression-like behaviors in rats by enhancing the BDNF/TrkB signaling pathway and inhibiting autophagy in the hippocampus ^116^. |
|  |  | SAMP8 and SAMR1 mice | M | Silibinin improves memory and learning deficits in SAMP8 mice by inhibiting microglial activation, reducing proinflammatory cytokines, and modulating MAPK pathways ^117^. |
|  |  | Sprague–Dawley rats injected with LPS | M | Silibinin protects against LPS-induced neuroinflammation and cognitive dysfunction in rats by reducing inflammatory cytokines, inhibiting NF-κB signaling, and activating the ROS–BDNF–TrkB pathway in the hippocampus ^118^. |
| Epigallocatechin-3-gallate (EGCG) (A powerful antioxidant found in green tea) | ^119-121^ | Sprague-Dawley (SD) rats injected with Aβ 25–35 | M | EGCG improved learning and memory in AD rats by reducing Tau hyperphosphorylation, downregulating *BACE1* and Aβ1-42 expression, enhancing the antioxidant system, and decreasing AchE activity, thereby increasing Ach levels ^122^. |
|  |  | SAMP8 and SAMR1 mice |  | EGCG, a polyphenol from green tea, acts as an HDAC inhibitor that upregulates *NEP* expression, enhances Aβ degradation, reduces amyloid burden, and improves cognitive function in AD models ^123^ |
|  |  | APP/PS1 mice–Neuro-2a cells |  | EGCG alleviates Cu²⁺- and Zn²⁺-induced Aβ₄₀ aggregation and neurotoxicity by chelation, reducing ROS production, and disrupting Aβ oligomers and fibrils; notably, ECG crosses the BBB, reducing Aβ plaques and protecting neurons ^124^ |
|  |  | Cerebral spheroids derived from MensB-MenSCs (mesenchymal stem cells derived from menstrual blood) and induced to cholinergic-like neuronal cell lineage |  | EGCG reduces key pathological markers of FAD, including AβPP fragments, oxidized DJ-1, phosphorylated tau, mitochondrial dysfunction, and caspase-3 activation in mutant PSEN1 cortical spheroids ^125^. |
|  |  | Aged Swiss albino strain mice (normal aged and D-gal induced) | M | EGCG and curcumin co-administration significantly improved memory retention in D-gal and normal-aged mice, enhanced oxidative stress biomarkers, and offered superior neuroprotection compared to either compound alone ^126^. |
|  |  |  |  | EGCG induces tau fibril disaggregation by targeting a specific pharmacophore with charge-pairing and curvature changes driving fibril destabilization ^127^. |
|  |  | APP/PS1 mice |  | EGCG alleviates cognitive impairments in APP/PS1 mice by enhancing synaptic integrity, reducing Aβ plaques, and exerting anti-inflammatory effects ^128^ |
|  |  |  |  | EGCG disrupts Aβ42 protofibrils by forming key interactions, including hydrogen bonds and cation-π interactions, particularly involving the gallic acid ester group ^129^. |
| Kaempferol (A flavonoid found in various fruits and vegetables) | ^130-132^ | Wistar rats injected with STZ | M&F | Kaempferol treatment improved spatial memory and reduced oxidative stress, enhancing hippocampal levels of antioxidants (SOD and glutathione) and reducing malondialdehyde, in a rat AD model ^133^. |
|  |  | PC-12 cells |  | Kaempferol attenuates Aβ25–35-induced apoptosis in PC-12 cells by regulating the ER/ERK/MAPK signaling pathway ^134^. |
|  |  | C57BL/6 mice administered with D-gal/Alcl3 | M&F | Kaempferol improved cognitive and neurological function in d-galactose/aluminum chloride-induced mice by enhancing memory, reducing oxidative stress and inflammation, and modulating neurotransmitter levels and the SIRT1/NF-κB pathway ^135^. |
|  |  | *Drosophila melanogaster* |  | Kaempferol reduces oxidative stress, improves cognitive function, protects dopaminergic neurons, and enhances behavior in a Drosophila Parkinson’s disease model by scavenging free radicals and boosting tyrosine hydroxylase activity ^136^. |
| Quercetin (A flavonoid with potent antioxidant commonly found in fruits, vegetables, and grains) | ^137-140^ | Wistar rats injected with Aβ | M | Quercetin improved learning, memory, and adult neurogenesis in a rat model of AD by increasing neural stem cell proliferation, promoting neurogenesis-related gene expression, and enhancing hippocampal neurogenesis ^141^. |
|  |  | Wistar rats administered with AlCl_3_ | M | Quercetin-conjugated superparamagnetic iron oxide nanoparticles improved cognitive function, reduced oxidative stress, and modulated antioxidant, apoptotic, and *APP* gene expression more effectively than free quercetin in an AD rat model ^142^. |
|  |  | Wistar rats | M | Quercetin enhances RGC survival and function in glaucoma by boosting inhibitory GABAergic and reducing excitatory glutamatergic neurotransmission, alleviating excitotoxic damage ^143^. |
|  |  | SH-SY5Y cell line |  | Quercetin-modified gold-palladium  nanostructure efficiently induces autophagy in SH-SY5Y cells, promotes Aβ clearance, and protects cells from Aβ-induced toxicity, with high blood-brain barrier permeability and good biocompatibility ^144^ |
| Naringenin (A flavonoid found in citrus fruits) | ^145,146^ | HFD-fed SAMP8 mice |  | Naringenin improved spatial memory in Alzheimer's model mice by reducing Aβ, tau hyperphosphorylation, oxidative stress, and neuroinflammation ^147^. |
|  |  | A*β*_25–35_-injured C57BL/6J mice and A*β*_25–35_-injured PC12 cells | M | Naringin improved learning, memory, and neuronal survival in an Alzheimer's model by modulating ER, PI3K/AKT, and GSK-3β pathways, reducing Tau hyperphosphorylation, and exhibiting estrogen-like neuroprotection ^148^. |
|  |  | Wistar albino rats administered with Aβ + D-Gal | M | Naringin mitigated Aβ-induced cognitive impairment, cholinergic dysfunction, and mitochondrial toxicity in key brain regions via Mas receptor-mediated mechanisms ^149^ |
|  |  | APP/PS1 transgenic mice and BV2 microglia cell line | M | Naringenin improves cognitive function, reduced Aβ deposition, and alleviated neuroinflammation in APP/PS1 mice by inhibiting the MAPK signaling pathway^150^. |
|  |  | Wistar rats underwent Aβ microinjection surgery | M | Naringin and exercise improved spatial learning and memory in Aβ-injected rats by enhancing H₂S production, reducing inflammation, and exerting anti-apoptotic effects ^151^. |
|  |  | Aβ25-35 was used to induce an Alzheimer's disease model in SK-N-AS cells. |  | Hesperidin and naringin reduced Aβ and α-synuclein accumulation in an AD cell model, with naringin showing greater efficacy in decreasing Aβ and tau protein levels ^152^ |
|  |  | Swiss albino mice injected with Scopolamine | M | A naringin nano formulation demonstrated significant neuroprotective and anti-neuroinflammatory effects in an AD rat model, improving cognitive function, reducing oxidative stress, and modulating neuroinflammatory markers ^153^. |
|  |  | AlCl_3_-recived Albino rats | M | Naringin alleviates AlCl3-induced cognitive and motor impairments in an AD rat model by reducing oxidative stress, lipid peroxidation, and tau accumulation while restoring glutathione and autophagic activity ^154^. |
|  |  | Sprague-Dawley (SD) rats injected with Aβ solution into the hippocampus. | M | Naringin improves learning, memory, and cognition in an Aβ-induced AD rat model by enhancing synaptic plasticity, reducing neuronal inflammation and apoptosis, and modulating the BDNF/TrkB/CREB signaling pathway ^155^. |
|  |  | Hydrocortisone-induced mic | M | Naringin improves cognitive deficits in hydrocortisone-induced mice by modulating Aβ metabolism, Tau hyperphosphorylation, neurotransmitter systems, oxidative stress, and apoptosis, possibly via ER interaction and the MAPK/P38 pathway ^156^. |
| Berberine (A bioactive compound extracted from several plants) | ^157-161^ | TgCRND8 mice |  | Berberine treatment in TgCRND8 mice reduces Aβ plaque load, glial activation, and cognitive impairment by regulating APP processing through the PI3K/Akt/GSK3 signaling pathway ^162^ |
|  |  | 3 × Tg-AD mice- Primary hippocampal neurons culture | M&F | Berberine promotes Aβ clearance, inhibits Aβ production, and improves memory in a 3 ×Tg-AD mouse model by enhancing autophagy through the PI3K/beclin-1 pathway and reducing BACE1 levels ^163^ |
|  |  | 3 × Tg-AD mice–Primary hippocampal neurons culture | M | Berberine improves cognitive deficits in a 3 × Tg AD mouse model by inhibiting the PERK/eIF2α signaling pathway, reducing BACE1-mediated Aβ production, and attenuating ER and oxidative stress ^164^. |
|  |  | 3 × Tg AD mice–Primary hippocampal neurons culture | M | Berberine improves spatial learning and memory in 3×Tg AD mice by attenuating tau hyperphosphorylation through the Akt/GSK-3β and protein phosphatase 2A pathways, and enhancing tau autophagic clearance via the PI3K/beclin-1 pathway ^165^ |
|  |  | B6C3-Tg transgenic mice | M&F | The combined berberine and curcumin treatment improved cognitive function in an AD, reducing Aβ, inflammation, oxidative stress, and enhancing autophagy through AMPK activation, outperforming single drugs ^166^. |
|  |  | 3×Tg AD mice | M | Berberine improved cognitive function in a triple-transgenic Alzheimer’s mouse model by reducing Aβ accumulation, inhibiting neuronal apoptosis, promoting angiogenesis, and enhancing cerebral blood flow ^167^. |
|  |  | Aβ-treated BV2 and N2a cells |  | Berberine promoted cell viability and inhibited apoptosis in Aβ-treated BV2 and N2a cells by regulating the miR-188/NOS1 axis ^168^. |
|  |  | STZ and Aβ25–35 induced Sprague–Dawley rats | M | Berberine alleviated cognitive deficits, reduced blood glucose and lipid levels, protected neurons, and suppressed ER stress in a rat model of combined T2D and AD ^169^ |
|  |  | Primary microglial and BV2 cells |  | Berberine inhibits neuroinflammation in primary microglial and BV2 cells by suppressing NF-κB activation and downregulating pro-inflammatory markers ^170^ |
|  |  | N2a/APP695sw and N2a cells culture |  | Berberine reduces Aβ generation and decreases BACE1 expression by activating AMPK in neuroblastoma cells and primary neurons ^171^ |
|  |  | APP/PS1 mice |  | Berberine improves learning and memory deficits in APP/PS1 mice by reducing Aβ levels, inhibiting β/γ-secretases, enhancing α-secretases, and lowering hippocampal Aβ levels ^172^. |
|  |  | APP/PS1 mice | M | berberine improves cognitive function in APP/PS1 mice by reducing hyperphosphorylated tau, inhibiting NF-κB signaling, and alleviating oxidative stress and neuroinflammation ^173^. |
|  |  | APP/PS1 transgenic mice | M | Berberine alleviates cognitive impairment and reduces key AD pathologies by suppressing ER stress, inhibiting the GSK3β and PERK/eIF2α/BACE1 signaling pathways, and lowering tau phosphorylation and Aβ42 production ^174^. |
| Genistein (A soy-derived isoflavonoid) | ^175^ | Sprague-Dawley rats administered with D-galactose+Aβ_25-35_ | M&F | Genistein protects against Alzheimer's by enhancing memory, preserving hippocampal neurons, and inhibiting ER stress-mediated apoptosis via downregulation of GRP78, CHOP, Caspase-12, and PERK ^176^. |
|  |  | SH-SY5Y cells |  | Genistein protects against Aβ-induced neurotoxicity by reducing apoptosis, preventing Akt inactivation, and inhibiting Tau hyperphosphorylation in differentiated SH-SY5Y cells ^177^. |
|  |  | *ApoE^−/−^* mouse fed an HFD | M | Genistein reduces neuroinflammation, oxidative stress, Aβ deposition, and tau hyperphosphorylation in ApoE−/− mice by modulating APP-processing enzymes and inactivating GSK-3β and JNK ^178^. |
|  |  | Wistar rats administered with Aβ_1–42_ peptide | M | Genistein improves Aβ1–42-induced cognitive impairment in rats by reducing synaptotoxicity, inhibiting Tau hyperphosphorylation, and inactivating ERK ^179^. |
|  |  | Wistar rats administered with Aβ_1–40_ | M | Genistein treatment inhibits Aβ1–40 aggregation, reduces neuronal damage, and alleviates astrogliosis in the hippocampus of rats ^180^. |
|  |  | Sprague-Dawley rats administered with D-galactose+Aβ_25-35_ | F | Genistein reduces tau hyperphosphorylation by regulating CAMK4 in an AD rat model, improving cognitive function and alleviating hippocampal neuronal damage ^181^. |
|  |  | Sprague-Dawley rats administered with D-galactose+Aβ_25-35_ | F | Genistein protects against Aβ toxicity in Alzheimer's by inhibiting the mitochondrial apoptotic pathway, reducing apoptosis, and improving memory ^182^. |
|  |  | Randomized, double-blind, placebo-controlled phase 2 clinical study-27 patients | M&F | Genistein treatment improved cognitive function in prodromal Alzheimer's patients and prevented increased Aβ deposition in the anterior cingulate gyrus ^183^ |
|  |  | Primary cultures of rat cortical astrocytes |  | Genistein, like estradiol, prevents Aβ-induced inflammation in astrocytes by upregulating PPAR-γ expression ^184^. |
|  |  | Wistar rats administered with STZ | M | High-dose genistein activates autophagy, leading to the degradation of Aβ and tau proteins, normalizing behavior and biochemical defects in a rat model of AD ^185^. |
| Caffeic acid (A naturally occurring polyphenolic compound found in various plants) | ^146,186^ | A53T transgenic mice–  SH-SY5Y cells | M | Caffeic acid alleviated A53T α-synuclein-induced neurotoxicity by activating the JNK/Bcl-2 autophagy pathway, reducing aggregation, and protecting dopaminergic neurons in a Parkinson’s disease mouse model ^187^. |
|  |  | ApoE^−/−^ mice | F | Caffeic acid reduced atherosclerotic lesions and cognitive decline in ApoE−/− mice, linked to elevated HDL-c, upregulated *ABCA1* and *ABCG1*, and reduced inflammation and pro-inflammatory cytokines ^188^. |
|  |  |  |  | Caffeic acid inhibits Aβ1-42 aggregation and disrupts mature fibrils in aqueous solutions, with its disaggregating activity slightly reduced by the presence of lipid membranes ^189^ |
|  |  | Sprague–Dawley rats fed a HF diet | M | Caffeic acid improves memory and learning in hyperinsulinemic rats, enhanced antioxidant activity, reduced Aβ accumulation, and promoted neuroprotection by modulating key signaling pathways ^190^ |
| Emodin (A naturally occurring anthraquinone compound) | ^191-193^ | APP/PS1 transgenic mice– HT22 mouse hippocampal neurons | M | Emodin significantly improves cognitive function, reduced amyloid plaque accumulation, protected hippocampal neurons from Aβ1-42 toxicity, and enhanced PKC phosphorylation ^194^. |
|  |  | Sprague-Dawley rats treated with homocysteine injection | M | Emodin (80 mg/kg/day) improved cognition in hyperhomocysteinemic rats by reducing Aβ and tau phosphorylation, restoring synaptic proteins and neurons, and suppressing inflammation and oxidative stress ^195^. |
|  |  | APP/PS1 double-transgenic mice | M | Emodin protects against Aβ-induced apoptosis in U251 cells and Aβ deposition in APP/PS1 mice by enhancing Nrf2-mediated antioxidant activity, improving mitochondrial function, reducing oxidative stress, and enhancing learning and memory ^196^. |
|  |  | APP/PS1 double-transgenic mice | M&F | Emodin inhibits Aβ42 aggregation, reduces Aβ-induced cytotoxicity, and improves cognitive function in AD mice ^197^. |
|  |  | Sprague–Dawley rats received AlCl_3_ | M | Aloe emodin improves cognitive function in aluminum-induced AD rats by reducing aluminum burden, decreasing Aβ42 deposition, regulating the cholinergic system, and suppressing neuroinflammation ^198^. |
|  |  | PC12 cell |  | Emodin protects against Aβ25-35-induced toxicity in PC12 cells by reducing oxidative stress, inhibiting ferroptosis, and suppressing neuroinflammation through the Nrf2/GPX4 and TLR4/P-NF-κB/NLRP3 pathways ^199^ |
|  |  | PC12 cells |  | Emodin protects PC12 cells from oxidative stress-induced apoptosis by inhibiting PKM2 activity, promoting its dimerization, and activating the PKM2/Nrf2/ARE pathway ^200^. |
|  |  | BV2 Cell Culture–cell line of mouse hippocampus neurons HT-22 |  | Suppressing neuroinflammation and neuronal apoptosis by inhibiting NLRP3 inflammasome-induced pyroptosis in microglial cells and reducing neurotoxic effects in hippocampal neurons ^201^. |
| Celastrol (a triterpenoid compound derived from the plant *Tripterygium wilfordii*) | ^202-204^ | P301S Tau transgenic mice–3xTg mice–HeLa, N2a, and HEK293 cells | M&F–F | Celastrol enhances TFEB-mediated autophagy, promotes Tau degradation, and improves memory and cognitive deficits in Alzheimer's models ^205^. |
|  |  | Sprague-Dawley treated with HFD and STZ | M | Celastrol improves insulin sensitivity, reduces inflammation, enhances synaptic plasticity, and alleviates cognitive deficits in diabetic rats by modulating the insulin signaling pathway and lowering Aβ levels ^201^. |
|  |  | C57BL/6 injected with MPTP | M | Celastrol protects dopamine neurons and alleviates motor deficits in Parkinson's disease by inhibiting the NLRP3 inflammasome and modulating the Nrf2-NLRP3-caspase-1 pathway ^206^. |
| TEPP-46 (A small molecule inhibitor that selectively targets and inhibits the nuclear translocation of PKM2) | ^207^ | Peritoneal macrophages and Kupffer cells from C57BL/6 mice |  | TEPP-46 promotes macrophage endotoxin tolerance by enhancing mitochondrial biogenesis through the activation of PKM2 tetramers and upregulating key mitochondrial regulators like *PGC-1α, NRF1/2,* and *mtTFA*, thereby reducing inflammatory cytokine release ^208^. |
|  |  | Primary astrocytes  Originated from Experimental autoimmune encephalomyelitis C57BL/6 mice model | F | TEPP-46 mitigates astrocyte activation and proliferation by preventing PKM2 nuclear translocation and suppressing glycolysis, highlighting its potential as a therapeutic target for CNS diseases ^209^. |
|  |  | 3xTg mice–Brain samples from AD patients–HEK293T cells– neuronal cells and astrocytes isolated from mouse primary cortex |  | TEPP-46was found to reduce the stimulatory effect of PKM2 on γ-secretase activity ^210^. |
|  |  | LPS-induced C57BL/6 mice– BV2 cells | M | TEPP-46 prevented PKM2 nuclear translocation, reducing LPS-induced microglial inflammation and pyroptosis by disrupting PKM2’s interaction with ATF2 ^211^. |
| Compound 3K (PKM2-IN-1) | ^212-214^ | 5XFAD-Mouse microglial BV2 cell | M&F | Compound 3K, a PKM2 inhibitor, significantly reduced Aβ plaque content, reactive microgliosis, and pro-inflammatory factors in 5XFAD mice ^215^. |
|  |  | Primary astrocytes were derived from an inflammatory pain Sprague-Dawley rat model | M | PKM2-IN-1 inhibits PKM2-mediated glycolysis, reducing astrocyte activation, lactate production, and inflammatory markers in complete Freund’s adjuvant (CFA)- and LPS-induced models ^216^ |
| DASA-58 | ^207^ | Traumatic Brain Injury C57BL/6 mice model | M&F | DASA-58 attenuates glycolysis and facilitates the transition of microglia to an anti-inflammatory phenotype ^217^. |
|  |  | Primary astrocytes  Originated from Experimental autoimmune encephalomyelitis C57BL/6 mouse model | F | 2-DASA-58 mitigates astrocyte glycolysis and activation ^209^. |

**Supplementary references**

1 Zhao, X. *et al.* Shikonin Inhibits Tumor Growth in Mice by Suppressing Pyruvate Kinase M2-mediated Aerobic Glycolysis. *Sci Rep* **8**, 14517 (2018). <https://doi.org/10.1038/s41598-018-31615-y>

2 Chen, J. *et al.* Shikonin and its analogs inhibit cancer cell glycolysis by targeting tumor pyruvate kinase-M2. *Oncogene* **30**, 4297-4306 (2011). <https://doi.org/10.1038/onc.2011.137>

3 Li, D. & Wang, M. Shikonin Attenuate Behavioral Defects, Oxidative Stress, and Neuroinflammation During Aluminum Chloride-induced| Alzheimer’s Disease Condition in an in-vivo Experimental Model. *Pharmacognosy Magazine*, 09731296241239153 (2024).

4 Tong, Y. *et al.* Shikonin Protects PC12 Cells Against beta-amyloid Peptide-Induced Cell Injury Through Antioxidant and Antiapoptotic Activities. *Sci Rep* **8**, 26 (2018). <https://doi.org/10.1038/s41598-017-18058-7>

5 Zyuz'kov, G. N., Miroshnichenko, L. A. e., Polyakova, T. Y. & Simanina, E. V. Neuroprotective and neuroregenerative effects of shikonin-mediated inhibition of NF-κB/Stat3 in alcoholic encephalopathy. *Letters in drug design & discovery* **20**, 2045-2054 (2023).

6 Ni, J. *et al.* Systemic administration of Shikonin ameliorates cognitive impairment and neuron damage in NPSLE mice. *J Neuroimmunol* **382**, 578166 (2023). <https://doi.org/10.1016/j.jneuroim.2023.578166>

7 Chen, D. B. *et al.* Quinones as preventive agents in Alzheimer's diseases: focus on NLRP3 inflammasomes. *J Pharm Pharmacol* **72**, 1481-1490 (2020). <https://doi.org/10.1111/jphp.13332>

8 Yang, Y. *et al.* Shikonin attenuates cerebral ischemia/reperfusion injury via inhibiting NOD2/RIP2/NF-kappaB-mediated microglia polarization and neuroinflammation. *J Stroke Cerebrovasc Dis* **33**, 107689 (2024). <https://doi.org/10.1016/j.jstrokecerebrovasdis.2024.107689>

9 Prasad, R. G., Choi, Y. H. & Kim, G. Y. Shikonin Isolated from Lithospermum erythrorhizon Downregulates Proinflammatory Mediators in Lipopolysaccharide-Stimulated BV2 Microglial Cells by Suppressing Crosstalk between Reactive Oxygen Species and NF-kappaB. *Biomol Ther (Seoul)* **23**, 110-118 (2015). <https://doi.org/10.4062/biomolther.2015.006>

10 Li, Q., Zeng, J., Su, M., He, Y. & Zhu, B. Acetylshikonin from Zicao attenuates cognitive impairment and hippocampus senescence in d-galactose-induced aging mouse model via upregulating the expression of SIRT1. *Brain Res Bull* **137**, 311-318 (2018). <https://doi.org/10.1016/j.brainresbull.2018.01.007>

11 Yao, M. *et al.* Shikonin inhibits neuronal apoptosis via regulating endoplasmic reticulum stress in the rat model of double-level chronic cervical cord compression. *Cell Biol Toxicol* **39**, 907-928 (2023). <https://doi.org/10.1007/s10565-021-09648-3>

12 Wang, Y. *et al.* Acetylshikonin, a Novel AChE Inhibitor, Inhibits Apoptosis via Upregulation of Heme Oxygenase-1 Expression in SH-SY5Y Cells. *Evid Based Complement Alternat Med* **2013**, 937370 (2013). <https://doi.org/10.1155/2013/937370>

13 Venkatramani, A., Mukherjee, S., Kumari, A. & Panda, D. Shikonin impedes phase separation and aggregation of tau and protects SH-SY5Y cells from the toxic effects of tau oligomers. *Int J Biol Macromol* **204**, 19-33 (2022). <https://doi.org/10.1016/j.ijbiomac.2022.01.172>

14 Greuel, B. K., Da Silva, D. E., Robert-Gostlin, V. N. & Klegeris, A. Natural Compounds Oridonin and Shikonin Exhibit Potentially Beneficial Regulatory Effects on Select Functions of Microglia. *Brain Sci* **14** (2024). <https://doi.org/10.3390/brainsci14040328>

15 Sha, S., Jin, N., Zhou, R., Ruan, Y. & Ouyang, Y. The Activation of PKM2 Induces Pyroptosis in Hippocampal Neurons via the NLRP3/Caspase-1/GSDMD Pathway in Neonatal Rats With Hypoxic-Ischemic Brain Injury. *Brain Behav* **14**, e70108 (2024). <https://doi.org/10.1002/brb3.70108>

16 Iqbal, M. A. & Bamezai, R. N. Resveratrol inhibits cancer cell metabolism by down regulating pyruvate kinase M2 via inhibition of mammalian target of rapamycin. *PLoS One* **7**, e36764 (2012). <https://doi.org/10.1371/journal.pone.0036764>

17 Zhao, H. *et al.* Resveratrol induces apoptosis in human melanoma cell through negatively regulating Erk/PKM2/Bcl-2 axis. *OncoTargets and therapy*, 8995-9006 (2018).

18 Wu, H. *et al.* Resveratrol inhibits VEGF‐induced angiogenesis in human endothelial cells associated with suppression of aerobic glycolysis via modulation of PKM 2 nuclear translocation. *Clinical and Experimental Pharmacology and Physiology* **45**, 1265-1273 (2018).

19 Wu, H. *et al.* Resveratrol Induces Cancer Cell Apoptosis through MiR-326/PKM2-Mediated ER Stress and Mitochondrial Fission. *J Agric Food Chem* **64**, 9356-9367 (2016). <https://doi.org/10.1021/acs.jafc.6b04549>

20 Gu, J. *et al.* Neuroprotective Effect of Trans-Resveratrol in Mild to Moderate Alzheimer Disease: A Randomized, Double-Blind Trial. *Neurol Ther* **10**, 905-917 (2021). <https://doi.org/10.1007/s40120-021-00271-2>

21 Broderick, T. L. *et al.* Neuroprotective Effects of Chronic Resveratrol Treatment and Exercise Training in the 3xTg-AD Mouse Model of Alzheimer's Disease. *Int J Mol Sci* **21** (2020). <https://doi.org/10.3390/ijms21197337>

22 Zhu, C. W. *et al.* A randomized, double-blind, placebo-controlled trial of resveratrol with glucose and malate (RGM) to slow the progression of Alzheimer's disease: A pilot study. *Alzheimers Dement (N Y)* **4**, 609-616 (2018). <https://doi.org/10.1016/j.trci.2018.09.009>

23 Wang, H., Jiang, T., Li, W., Gao, N. & Zhang, T. Resveratrol attenuates oxidative damage through activating mitophagy in an in vitro model of Alzheimer's disease. *Toxicol Lett* **282**, 100-108 (2018). <https://doi.org/10.1016/j.toxlet.2017.10.021>

24 Karuppagounder, S. S. *et al.* Dietary supplementation with resveratrol reduces plaque pathology in a transgenic model of Alzheimer's disease. *Neurochem Int* **54**, 111-118 (2009). <https://doi.org/10.1016/j.neuint.2008.10.008>

25 Porquet, D. *et al.* Dietary resveratrol prevents Alzheimer's markers and increases life span in SAMP8. *Age (Dordr)* **35**, 1851-1865 (2013). <https://doi.org/10.1007/s11357-012-9489-4>

26 Moussa, C. *et al.* Resveratrol regulates neuro-inflammation and induces adaptive immunity in Alzheimer's disease. *J Neuroinflammation* **14**, 1 (2017). <https://doi.org/10.1186/s12974-016-0779-0>

27 Marambaud, P., Zhao, H. & Davies, P. Resveratrol promotes clearance of Alzheimer's disease amyloid-beta peptides. *J Biol Chem* **280**, 37377-37382 (2005). <https://doi.org/10.1074/jbc.M508246200>

28 Porquet, D. *et al.* Neuroprotective role of trans-resveratrol in a murine model of familial Alzheimer's disease. *J Alzheimers Dis* **42**, 1209-1220 (2014). <https://doi.org/10.3233/JAD-140444>

29 Yang, L., Wang, Y., Zheng, G., Li, Z. & Mei, J. Resveratrol-loaded selenium/chitosan nano-flowers alleviate glucolipid metabolism disorder-associated cognitive impairment in Alzheimer's disease. *Int J Biol Macromol* **239**, 124316 (2023). <https://doi.org/10.1016/j.ijbiomac.2023.124316>

30 Abozaid, O. A. R. *et al.* Resveratrol-Selenium Nanoparticles Alleviate Neuroinflammation and Neurotoxicity in a Rat Model of Alzheimer's Disease by Regulating Sirt1/miRNA-134/GSK3beta Expression. *Biol Trace Elem Res* **200**, 5104-5114 (2022). <https://doi.org/10.1007/s12011-021-03073-7>

31 Al-Edresi, S., Alsalahat, I., Freeman, S., Aojula, H. & Penny, J. Resveratrol-mediated cleavage of amyloid β1–42 peptide: potential relevance to Alzheimer's disease. *Neurobiology of Aging* **94**, 24-33 (2020).

32 Rao, Y. L. *et al.* Comparison of malondialdehyde levels and superoxide dismutase activity in resveratrol and resveratrol/donepezil combination treatment groups in Alzheimer's disease induced rat model. *3 Biotech* **11**, 329 (2021). <https://doi.org/10.1007/s13205-021-02879-5>

33 Li, C., Wang, N., Zheng, G. & Yang, L. Oral administration of resveratrol-selenium-peptide nanocomposites alleviates Alzheimer’s disease-like pathogenesis by inhibiting Aβ aggregation and regulating gut microbiota. *ACS Applied Materials & Interfaces* **13**, 46406-46420 (2021).

34 Bartra, C. *et al.* Resveratrol Activates Antioxidant Protective Mechanisms in Cellular Models of Alzheimer's Disease Inflammation. *Antioxidants (Basel)* **13** (2024). <https://doi.org/10.3390/antiox13020177>

35 Labban, S., Alghamdi, B. S., Alshehri, F. S. & Kurdi, M. Effects of melatonin and resveratrol on recognition memory and passive avoidance performance in a mouse model of Alzheimer's disease. *Behav Brain Res* **402**, 113100 (2021). <https://doi.org/10.1016/j.bbr.2020.113100>

36 Ma, X. *et al.* Neuroprotective Effect of Resveratrol via Activation of Sirt1 Signaling in a Rat Model of Combined Diabetes and Alzheimer's Disease. *Front Neurosci* **13**, 1400 (2019). <https://doi.org/10.3389/fnins.2019.01400>

37 Foudah, A. I., Devi, S., Alam, A., Salkini, M. A. & Ross, S. A. Anticholinergic effect of resveratrol with vitamin E on scopolamine-induced Alzheimer's disease in rats: Mechanistic approach to prevent inflammation. *Front Pharmacol* **14**, 1115721 (2023). <https://doi.org/10.3389/fphar.2023.1115721>

38 Tao, G. *et al.* Dihydro-resveratrol ameliorates NLRP3 inflammasome-mediated neuroinflammation via Bnip3-dependent mitophagy in Alzheimer's disease. *Br J Pharmacol* **182**, 1005-1024 (2025). <https://doi.org/10.1111/bph.17373>

39 Agarwal, T. *et al.* Oxyresveratrol-β-cyclodextrin mitigates streptozotocin-induced Alzheimer's model cognitive impairment, histone deacetylase activity in rats: in silico & in vivo studies. *Scientific Reports* **14**, 9897 (2024).

40 Hao, Y. *et al.* Resveratrol and Sir2 Reverse Sleep and Memory Defects Induced by Amyloid Precursor Protein. *Neurosci Bull* **39**, 1117-1130 (2023). <https://doi.org/10.1007/s12264-023-01056-3>

41 Pal, K. *et al.* A pro-oxidant combination of resveratrol and copper down-regulates multiple biological hallmarks of ageing and neurodegeneration in mice. *Sci Rep* **12**, 17209 (2022). <https://doi.org/10.1038/s41598-022-21388-w>

42 Jhanji, M. *et al.* Cis- and trans-resveratrol have opposite effects on histone serine-ADP-ribosylation and tyrosine induced neurodegeneration. *Nat Commun* **13**, 3244 (2022). <https://doi.org/10.1038/s41467-022-30785-8>

43 Shan, S. *et al.* Apigenin Restrains Colon Cancer Cell Proliferation via Targeted Blocking of Pyruvate Kinase M2-Dependent Glycolysis. *J Agric Food Chem* **65**, 8136-8144 (2017). <https://doi.org/10.1021/acs.jafc.7b02757>

44 Sun, T. *et al.* Apigenin intervenes in liver fibrosis by regulating PKM2-HIF-1α mediated oxidative stress. *Biochemical and Biophysical Research Communications* **721**, 150130 (2024).

45 Shi, J. *et al.* The interaction between apigenin and PKM2 restrains progression of colorectal cancer. *J Nutr Biochem* **121**, 109430 (2023). <https://doi.org/10.1016/j.jnutbio.2023.109430>

46 Fang, M. *et al.* Insights into Molecular Mechanisms of EGCG and Apigenin on Disrupting Amyloid-Beta Protofibrils Based on Molecular Dynamics Simulations. *J Phys Chem B* **126**, 8155-8165 (2022). <https://doi.org/10.1021/acs.jpcb.2c04230>

47 Yarim, G. F. *et al.* Apigenin alleviates neuroinflammation in a mouse model of Parkinson's disease. *Int J Neurosci*, 1-10 (2022). <https://doi.org/10.1080/00207454.2022.2089136>

48 Chesworth, R. *et al.* Spatial Memory and Microglia Activation in a Mouse Model of Chronic Neuroinflammation and the Anti-inflammatory Effects of Apigenin. *Front Neurosci* **15**, 699329 (2021). <https://doi.org/10.3389/fnins.2021.699329>

49 Ahmedy, O. A., Abdelghany, T. M., El-Shamarka, M. E. A., Khattab, M. A. & El-Tanbouly, D. M. Apigenin attenuates LPS-induced neurotoxicity and cognitive impairment in mice via promoting mitochondrial fusion/mitophagy: role of SIRT3/PINK1/Parkin pathway. *Psychopharmacology (Berl)* **239**, 3903-3917 (2022). <https://doi.org/10.1007/s00213-022-06262-x>

50 Kim, Y., Kim, J., He, M., Lee, A. & Cho, E. Apigenin Ameliorates Scopolamine-Induced Cognitive Dysfunction and Neuronal Damage in Mice. *Molecules* **26** (2021). <https://doi.org/10.3390/molecules26175192>

51 Chiu, Y. J. *et al.* A Neuroprotective Action of Quercetin and Apigenin through Inhibiting Aggregation of Abeta and Activation of TRKB Signaling in a Cellular Experiment. *Biomol Ther (Seoul)* **31**, 285-297 (2023). <https://doi.org/10.4062/biomolther.2022.136>

52 Alsadat, A. M. *et al.* GSK-3beta as a target for apigenin-induced neuroprotection against Abeta 25-35 in a rat model of Alzheimer's disease. *Neuropeptides* **90**, 102200 (2021). <https://doi.org/10.1016/j.npep.2021.102200>

53 Dourado, N. S. *et al.* Neuroimmunomodulatory and Neuroprotective Effects of the Flavonoid Apigenin in in vitro Models of Neuroinflammation Associated With Alzheimer's Disease. *Front Aging Neurosci* **12**, 119 (2020). <https://doi.org/10.3389/fnagi.2020.00119>

54 Che, D. N. *et al.* Effect of Luteolin and Apigenin on the Production of Il-31 and Il-33 in Lipopolysaccharides-Activated Microglia Cells and Their Mechanism of Action. *Nutrients* **12** (2020). <https://doi.org/10.3390/nu12030811>

55 Chiang, N. N. *et al.* Flavones 7,8-DHF, Quercetin, and Apigenin Against Tau Toxicity via Activation of TRKB Signaling in DeltaK280 Tau(RD)-DsRed SH-SY5Y Cells. *Front Aging Neurosci* **13**, 758895 (2021). <https://doi.org/10.3389/fnagi.2021.758895>

56 Kurniati, D., Hirai, S. & Egashira, Y. Effect of apigenin on tryptophan metabolic key enzymes expression in lipopolysaccharide-induced microglial cells and its mechanism. *Heliyon* **9**, e12743 (2023). <https://doi.org/10.1016/j.heliyon.2022.e12743>

57 Jameie, S. B. *et al.* beta-Amyloid Formation, Memory, and Learning Decline Following Long-term Ovariectomy and Its Inhibition by Systemic Administration of Apigenin and beta-Estradiol. *Basic Clin Neurosci* **12**, 383-394 (2021). <https://doi.org/10.32598/bcn.2021.2634.1>

58 Islam, M. A. *et al.* Evaluation of cholinesterase inhibitory and antioxidant activity of Wedelia chinensis and isolation of apigenin as an active compound. *BMC Complement Med Ther* **21**, 204 (2021). <https://doi.org/10.1186/s12906-021-03373-4>

59 Siddique, Y. H. *et al.* Beneficial effects of apigenin on the transgenic Drosophila model of Alzheimer's disease. *Chem Biol Interact* **366**, 110120 (2022). <https://doi.org/10.1016/j.cbi.2022.110120>

60 Kim, Y. J., Cho, E. J., Lee, A. Y. & Seo, W. T. Apigenin ameliorates oxidative stress-induced neuronal apoptosis in SH-SY5Y cells. (2021).

61 Siddiqui, F. A. *et al.* Curcumin decreases Warburg effect in cancer cells by down-regulating pyruvate kinase M2 via mTOR-HIF1α inhibition. *Scientific reports* **8**, 8323 (2018).

62 Mojzes, A. *et al.* Cell-Type Specific Metabolic Response of Cancer Cells to Curcumin. *Int J Mol Sci* **21** (2020). <https://doi.org/10.3390/ijms21051661>

63 Sarfraz, I., Rasul, A., Jabeen, F., Sultana, T. & Adem, S. Identification of Natural Compounds as Inhibitors of Pyruvate Kinase M2 for Cancer Treatment. *Molecules* **27** (2022). <https://doi.org/10.3390/molecules27207113>

64 Yan, D. *et al.* Functionalized curcumin/ginsenoside Rb1 dual-loaded liposomes: Targeting the blood-brain barrier and improving pathological features associated in APP/PS-1 mice. *Journal of Drug Delivery Science and Technology* **86**, 104633 (2023).

65 Qiao, P. *et al.* Curcumin prevents neuroinflammation by inducing microglia to transform into the M2-phenotype via CaMKKβ-dependent activation of the AMP-activated protein kinase signal pathway. *Current Alzheimer Research* **17**, 735-752 (2020).

66 Yang, F. *et al.* Curcumin inhibits formation of amyloid beta oligomers and fibrils, binds plaques, and reduces amyloid in vivo. *J Biol Chem* **280**, 5892-5901 (2005). <https://doi.org/10.1074/jbc.M404751200>

67 Rao, P., Goswami, D. & Rawal, R. M. Revealing the molecular interplay of curcumin as Culex pipiens Acetylcholine esterase 1 (AChE1) inhibitor. *Sci Rep* **11**, 17474 (2021). <https://doi.org/10.1038/s41598-021-96963-8>

68 Wang, H. M. *et al.* PPARgamma agonist curcumin reduces the amyloid-beta-stimulated inflammatory responses in primary astrocytes. *J Alzheimers Dis* **20**, 1189-1199 (2010). <https://doi.org/10.3233/JAD-2010-091336>

69 Bahrami, A., Jafari-Nozad, A. M., Karbasi, S., Ayadilord, M. & Ferns, G. A. Efficacy of Curcumin on Cognitive Function Scores in Women with Premenstrual Syndrome and Dysmenorrhea: A Triple-Blind, Placebo-Controlled Clinical Trial. *Chin J Integr Med* **29**, 387-393 (2023). <https://doi.org/10.1007/s11655-023-3732-3>

70 An, J. *et al.* Early onset diagnosis in Alzheimer's disease patients via amyloid-beta oligomers-sensing probe in cerebrospinal fluid. *Nat Commun* **15**, 1004 (2024). <https://doi.org/10.1038/s41467-024-44818-x>

71 Maruyama, H. *et al.* Long-term oral administration of curcumin is effective in preventing short-term memory deterioration and prolonging lifespan in a mouse model of Alzheimer’s disease. *Advances in Traditional Medicine* **24**, 373-385 (2024).

72 Li, G. *et al.* Curcumin reverses cognitive deficits through promoting neurogenesis and synapse plasticity via the upregulation of PSD95 and BDNF in mice. *Sci Rep* **15**, 1135 (2025). <https://doi.org/10.1038/s41598-024-82571-9>

73 Wu, X. *et al.* A network pharmacology approach to identify the mechanisms and molecular targets of curcumin against Alzheimer disease. *Medicine (Baltimore)* **101**, e30194 (2022). <https://doi.org/10.1097/MD.0000000000030194>

74 Di Martino, R. M. C. *et al.* Novel Curcumin-Diethyl Fumarate Hybrid as a Dualistic GSK-3beta Inhibitor/Nrf2 Inducer for the Treatment of Parkinson's Disease. *ACS Chem Neurosci* **11**, 2728-2740 (2020). <https://doi.org/10.1021/acschemneuro.0c00363>

75 Zou, X. *et al.* Curcumin and homotaurine suppress amyloid-β25–35 aggregation in synthetic brain membranes. *ACS Chemical Neuroscience* **12**, 1395-1405 (2021).

76 Han, Y. *et al.* Curcumin improves memory deficits by inhibiting HMGB1-RAGE/TLR4-NF-kappaB signalling pathway in APPswe/PS1dE9 transgenic mice hippocampus. *J Cell Mol Med* **25**, 8947-8956 (2021). <https://doi.org/10.1111/jcmm.16855>

77 Jithavech, P. *et al.* Physicochemical investigation of a novel curcumin diethyl gamma-aminobutyrate, a carbamate ester prodrug of curcumin with enhanced anti-neuroinflammatory activity. *PLoS One* **17**, e0265689 (2022). <https://doi.org/10.1371/journal.pone.0265689>

78 Dai, Y. *et al.* Bis-iodine-labeled Curcumin as a Potential CT Imaging Agent for beta-amyloid Plaques in the Brain. *CNS Neurol Disord Drug Targets* **22**, 1120-1132 (2023). <https://doi.org/10.2174/1871527321666220707091435>

79 Yu, H. *et al.* Protective and anti-oxidative effects of curcumin and resveratrol on Abeta-oligomer-induced damage in the SH-SY5Y cell line. *J Neurol Sci* **441**, 120356 (2022). <https://doi.org/10.1016/j.jns.2022.120356>

80 Matsui, A. *et al.* Curcumin tautomerization in the mechanism of pentameric amyloid- beta42 oligomers disassembly. *Biochem Biophys Res Commun* **666**, 68-75 (2023). <https://doi.org/10.1016/j.bbrc.2023.04.076>

81 Kuang, S. *et al.* A Photolabile Curcumin-Diazirine Analogue Enables Phototherapy with Physically and Molecularly Produced Light for Alzheimer's Disease Treatment. *Angew Chem Int Ed Engl* **62**, e202312519 (2023). <https://doi.org/10.1002/anie.202312519>

82 Sallaberry, C. A. *et al.* Curcumin Reduces Amyloid Beta Oligomer Interactions with Anionic Membranes. *ACS Chem Neurosci* **14**, 4026-4038 (2023). <https://doi.org/10.1021/acschemneuro.3c00512>

83 Shao, S., Ye, X., Su, W. & Wang, Y. Curcumin alleviates Alzheimer's disease by inhibiting inflammatory response, oxidative stress and activating the AMPK pathway. *J Chem Neuroanat* **134**, 102363 (2023). <https://doi.org/10.1016/j.jchemneu.2023.102363>

84 Ermiş, M. & Çiftci, G. Role of curcumin on beta-amyloid protein, tau protein, and biochemical and oxidative changes in streptozotocin-induced diabetic rats. *Naunyn-Schmiedeberg's Archives of Pharmacology* **397**, 9833-9844 (2024).

85 Moosavi, M., Bagheri-Mohammadi, S., Firouzan, B., Javadpour, P. & Ghasemi, R. Nanocurcumin prevents memory impairment, hippocampal apoptosis, Akt and CaMKII-α signaling disruption in the central STZ model of Alzheimer’s disease in rat. *Behavioural Brain Research* **471**, 115129 (2024).

86 Shi, P. *et al.* Pharmacological effects and mechanisms of bee venom and its main components: Recent progress and perspective. *Frontiers in pharmacology* **13**, 1001553 (2022).

87 Fan, X.-g. *et al.* Melittin ameliorates inflammation in mouse acute liver failure via inhibition of PKM2-mediated Warburg effect. *Acta Pharmacologica Sinica* **42**, 1256-1266 (2021).

88 Huang, J.-Y. *et al.* Melittin suppresses epithelial–mesenchymal transition and metastasis in human gastric cancer AGS cells via regulating Wnt/BMP associated pathway. *Bioscience, Biotechnology, and Biochemistry* **85**, 2250-2262 (2021).

89 Xing, X. *et al.* Neuroprotective Effects of Melittin Against Cerebral Ischemia and Inflammatory Injury via Upregulation of MCPIP1 to Suppress NF-kappaB Activation In Vivo and In Vitro. *Neurochem Res* **49**, 348-362 (2024). <https://doi.org/10.1007/s11064-023-04030-7>

90 Sha, X., Lin, J., Wu, K., Lu, J. & Yu, Z. The TRPV1-PKM2-SREBP1 axis maintains microglial lipid homeostasis in Alzheimer's disease. *Cell Death Dis* **16**, 14 (2025). <https://doi.org/10.1038/s41419-024-07328-8>

91 Suh, J. Y. *et al.* Crucial Role of c‐Myc/Monocarboxylate Transporter 4 Signaling in Capsaicin Induced Apoptotic and Anti‐Warburg Effects in Hepatocellular Carcinoma. *Phytotherapy Research* **39**, 536-547 (2025).

92 Zhang, Q. *et al.* Capsaicin ameliorates inflammation in a TRPV1-independent mechanism by inhibiting PKM2-LDHA-mediated Warburg effect in sepsis. *Cell chemical biology* **29**, 1248-1259. e1246 (2022).

93 Wang, C., Huang, W., Lu, J., Chen, H. & Yu, Z. TRPV1-Mediated Microglial Autophagy Attenuates Alzheimer's Disease-Associated Pathology and Cognitive Decline. *Front Pharmacol* **12**, 763866 (2021). <https://doi.org/10.3389/fphar.2021.763866>

94 Xu, W. *et al.* Capsaicin reduces Alzheimer-associated tau changes in the hippocampus of type 2 diabetes rats. *PLoS One* **12**, e0172477 (2017). <https://doi.org/10.1371/journal.pone.0172477>

95 Liu, C. H. *et al.* The Associations between a Capsaicin-Rich Diet and Blood Amyloid-beta Levels and Cognitive Function. *J Alzheimers Dis* **52**, 1081-1088 (2016). <https://doi.org/10.3233/JAD-151079>

96 Chen, L. *et al.* Capsaicin Attenuates Amyloid-beta-Induced Synapse Loss and Cognitive Impairments in Mice. *J Alzheimers Dis* **59**, 683-694 (2017). <https://doi.org/10.3233/JAD-170337>

97 Grimm, M. O. W. *et al.* The impact of capsaicinoids on APP processing in Alzheimer's disease in SH-SY5Y cells. *Sci Rep* **10**, 9164 (2020). <https://doi.org/10.1038/s41598-020-66009-6>

98 Du, Y. *et al.* TRPV1 activation alleviates cognitive and synaptic plasticity impairments through inhibiting AMPAR endocytosis in APP23/PS45 mouse model of Alzheimer's disease. *Aging Cell* **19**, e13113 (2020). <https://doi.org/10.1111/acel.13113>

99 Cakir, M. *et al.* Neuroprotective effect of transient receptor potential Vanilloid 1 agonist capsaicin in Alzheimer's disease model induced with okadaic acid. *Int Immunopharmacol* **118**, 109925 (2023). <https://doi.org/10.1016/j.intimp.2023.109925>

100 Wang, J. *et al.* Capsaicin consumption reduces brain amyloid-beta generation and attenuates Alzheimer's disease-type pathology and cognitive deficits in APP/PS1 mice. *Transl Psychiatry* **10**, 230 (2020). <https://doi.org/10.1038/s41398-020-00918-y>

101 Sarfraz, I., Rasul, A., Jabeen, F., Sultana, T. & Adem, Ş. Identification of Natural Compounds as Inhibitors of Pyruvate Kinase M2 for Cancer Treatment. *Molecules* **27**, 7113 (2022).

102 Monteiro, F. & Shetty, S. S. Natural antioxidants as inhibitors of pyruvate kinase M2 in Warburg phenotypes. *Journal of Herbal Medicine* **42**, 100750 (2023).

103 Ye, X., Sun, Y., Xu, Y., Chen, Z. & Lu, S. Integrated in silico-in vitro discovery of lung cancer-related tumor pyruvate kinase M2 (PKM2) inhibitors. *Medicinal Chemistry* **12**, 613-620 (2016).

104 Wang, C. *et al.* Autophagy activated by silibinin contributes to glioma cell death via induction of oxidative stress-mediated BNIP3-dependent nuclear translocation of AIF. *Cell Death & Disease* **11**, 630 (2020).

105 Duan, S. *et al.* Silibinin inhibits acetylcholinesterase activity and amyloid beta peptide aggregation: a dual-target drug for the treatment of Alzheimer's disease. *Neurobiol Aging* **36**, 1792-1807 (2015). <https://doi.org/10.1016/j.neurobiolaging.2015.02.002>

106 Liu, P. *et al.* Inhibition of GluN2B pathway is involved in the neuroprotective effect of silibinin on streptozotocin-induced Alzheimer's disease models. *Phytomedicine* **109**, 154594 (2023). <https://doi.org/10.1016/j.phymed.2022.154594>

107 Shen, L., Liu, L., Li, X. Y. & Ji, H. F. Regulation of gut microbiota in Alzheimer's disease mice by silibinin and silymarin and their pharmacological implications. *Appl Microbiol Biotechnol* **103**, 7141-7149 (2019). <https://doi.org/10.1007/s00253-019-09950-5>

108 Huo, Q. *et al.* Biomimetic silibinin-loaded macrophage-derived exosomes induce dual inhibition of Abeta aggregation and astrocyte activation to alleviate cognitive impairment in a model of Alzheimer's disease. *Mater Sci Eng C Mater Biol Appl* **129**, 112365 (2021). <https://doi.org/10.1016/j.msec.2021.112365>

109 Pan, Q., Ban, Y. & Xu, L. Silibinin-Albumin Nanoparticles: Characterization and Biological Evaluation Against Oxidative Stress-Stimulated Neurotoxicity Associated with Alzheimer's Disease. *J Biomed Nanotechnol* **17**, 1123-1130 (2021). <https://doi.org/10.1166/jbn.2021.3038>

110 Liu, P. *et al.* Silibinin ameliorates STING-mediated neuroinflammation via downregulation of ferroptotic damage in a sporadic Alzheimer's disease model. *Arch Biochem Biophys* **744**, 109691 (2023). <https://doi.org/10.1016/j.abb.2023.109691>

111 Liu, P. *et al.* Silibinin ameliorates STZ-induced impairment of memory and learning by up- regulating insulin signaling pathway and attenuating apoptosis. *Physiol Behav* **213**, 112689 (2020). <https://doi.org/10.1016/j.physbeh.2019.112689>

112 Bai, D. *et al.* Natural silibinin modulates amyloid precursor protein processing and amyloid-beta protein clearance in APP/PS1 mice. *J Physiol Sci* **69**, 643-652 (2019). <https://doi.org/10.1007/s12576-019-00682-9>

113 Liu, X. *et al.* Oral administration of silibinin ameliorates cognitive deficits of Parkinson’s disease mouse model by restoring mitochondrial disorders in hippocampus. *Neurochemical research* **46**, 2317-2332 (2021).

114 Bai, D. *et al.* Antioxidative and Anti-Apoptotic Roles of Silibinin in Reversing Learning and Memory Deficits in APP/PS1 Mice. *Neurochem Res* **42**, 3439-3445 (2017). <https://doi.org/10.1007/s11064-017-2389-3>

115 Wei, P. *et al.* Silibinin Ameliorates Formaldehyde-Induced Cognitive Impairment by Inhibiting Oxidative Stress. *Oxid Med Cell Longev* **2022**, 5981353 (2022). <https://doi.org/10.1155/2022/5981353>

116 Song, X. *et al.* Silibinin ameliorates anxiety/depression-like behaviors in amyloid beta-treated rats by upregulating BDNF/TrkB pathway and attenuating autophagy in hippocampus. *Physiol Behav* **179**, 487-493 (2017). <https://doi.org/10.1016/j.physbeh.2017.07.023>

117 Jin, G. *et al.* Silibinin rescues learning and memory deficits by attenuating microglia activation and preventing neuroinflammatory reactions in SAMP8 mice. *Neurosci Lett* **629**, 256-261 (2016). <https://doi.org/10.1016/j.neulet.2016.06.008>

118 Song, X. *et al.* Protective Effect of Silibinin on Learning and Memory Impairment in LPS-Treated Rats via ROS-BDNF-TrkB Pathway. *Neurochem Res* **41**, 1662-1672 (2016). <https://doi.org/10.1007/s11064-016-1881-5>

119 Wei, R., Hackman, R. M., Wang, Y. & Mackenzie, G. G. Targeting glycolysis with epigallocatechin-3-gallate enhances the efficacy of chemotherapeutics in pancreatic cancer cells and xenografts. *Cancers* **11**, 1496 (2019).

120 Zhou, Y., Qin, L., Li, C., Zhu, D. & Liu, B. EGCG enhances antitumor effect of apatinib in nonsmall cell lung cancer by targeting VEGF signaling to inhibit glycolysis. *Drug Development Research* **85**, e22239 (2024).

121 Wei, R. *et al.* Suppressing glucose metabolism with epigallocatechin-3-gallate (EGCG) reduces breast cancer cell growth in preclinical models. *Food & function* **9**, 5682-5696 (2018).

122 Nan, S., Wang, P., Zhang, Y. & Fan, J. Epigallocatechin-3-gallate provides protection against Alzheimer’s disease-induced learning and memory impairments in rats. *Drug Design, Development and Therapy*, 2013-2024 (2021).

123 Chang, X. *et al.* (-)-Epigallocatechin-3-gallate attenuates cognitive deterioration in Alzheimer's disease model mice by upregulating neprilysin expression. *Exp Cell Res* **334**, 136-145 (2015). <https://doi.org/10.1016/j.yexcr.2015.04.004>

124 Chen, T. *et al.* Inhibition of Abeta aggregates in Alzheimer's disease by epigallocatechin and epicatechin-3-gallate from green tea. *Bioorg Chem* **105**, 104382 (2020). <https://doi.org/10.1016/j.bioorg.2020.104382>

125 Soto-Mercado, V., Mendivil-Perez, M., Velez-Pardo, C. & Jimenez-Del-Rio, M. Neuroprotective Effect of Combined Treatment with Epigallocatechin 3-Gallate and Melatonin on Familial Alzheimer's Disease PSEN1 E280A Cerebral Spheroids Derived from Menstrual Mesenchymal Stromal Cells. *J Alzheimers Dis* **99**, S51-S66 (2024). <https://doi.org/10.3233/JAD-220903>

126 Rahman, M. A. *et al.* Combination of epigallocatechin 3 gallate and curcumin improves D-galactose and normal-aging associated memory impairment in mice. *Sci Rep* **13**, 12681 (2023). <https://doi.org/10.1038/s41598-023-39919-4>

127 Seidler, P. M. *et al.* Structure-based discovery of small molecules that disaggregate Alzheimer's disease tissue derived tau fibrils in vitro. *Nat Commun* **13**, 5451 (2022). <https://doi.org/10.1038/s41467-022-32951-4>

128 Bao, J. *et al.* Epigallocatechin-3-gallate Alleviates Cognitive Deficits in APP/PS1 Mice. *Curr Med Sci* **40**, 18-27 (2020). <https://doi.org/10.1007/s11596-020-2142-z>

129 Zhan, C., Chen, Y., Tang, Y. & Wei, G. Green Tea Extracts EGCG and EGC Display Distinct Mechanisms in Disrupting Abeta(42) Protofibril. *ACS Chem Neurosci* **11**, 1841-1851 (2020). <https://doi.org/10.1021/acschemneuro.0c00277>

130 Wu, H. *et al.* Kaempferol Reverses Aerobic Glycolysis via miR-339-5p-Mediated PKM Alternative Splicing in Colon Cancer Cells. *J Agric Food Chem* **69**, 3060-3068 (2021). <https://doi.org/10.1021/acs.jafc.0c07640>

131 Wu, H. *et al.* Kaempferol can reverse the 5-Fu resistance of colorectal cancer cells by inhibiting PKM2-mediated glycolysis. *International Journal of Molecular Sciences* **23**, 3544 (2022).

132 Wu, H. *et al.* Kaempferol reverses aerobic glycolysis via miR-339-5p-mediated PKM alternative splicing in colon cancer cells. *Journal of Agricultural and Food Chemistry* **69**, 3060-3068 (2021).

133 Babaei, P., Eyvani, K. & Kouhestani, S. Sex-Independent Cognition Improvement in Response to Kaempferol in the Model of Sporadic Alzheimer's Disease. *Neurochem Res* **46**, 1480-1486 (2021). <https://doi.org/10.1007/s11064-021-03289-y>

134 Zhang, N. *et al.* Protective mechanism of kaempferol against Aβ25-35-mediated apoptosis of pheochromocytoma (PC-12) cells through the ER/ERK/MAPK signalling pathway. *Archives of Medical Science: AMS* **17**, 406 (2020).

135 Lin, H., Wang, X., Zhao, J. & Lin, Z. Protective effect of kaempferol against cognitive and neurological disturbances induced by d-galactose and aluminum chloride in mice. *Journal of Functional Foods* **100**, 105385 (2023).

136 Rahul, Naz, F., Jyoti, S. & Siddique, Y. H. Effect of kaempferol on the transgenic Drosophila model of Parkinson's disease. *Sci Rep* **10**, 13793 (2020). <https://doi.org/10.1038/s41598-020-70236-2>

137 Chen, L.-l. *et al.* Quercetin protects against LPS-induced lung injury in mice via SIRT1-mediated suppression of PKM2 nuclear accumulation. *European Journal of Pharmacology* **936**, 175352 (2022).

138 Chan, C.-Y. *et al.* Oral squamous cell carcinoma cells with acquired resistance to erlotinib are sensitive to anti-cancer effect of quercetin via pyruvate kinase M2 (PKM2). *Cells* **12**, 179 (2023).

139 Chen, X. *et al.* Quercetin alleviates liver fibrosis via regulating glycolysis of liver sinusoidal endothelial cells and neutrophil infiltration. *Biomolecules and Biomedicine* **24**, 1806 (2024).

140 Xu, W.-l. *et al.* Quercetin antagonizes glucose fluctuation induced renal injury by inhibiting aerobic glycolysis via HIF-1α/miR-210/ISCU/FeS pathway. *Frontiers in medicine* **8**, 656086 (2021).

141 Karimipour, M. *et al.* Quercetin promotes learning and memory performance concomitantly with neural stem/progenitor cell proliferation and neurogenesis in the adult rat dentate gyrus. *Int J Dev Neurosci* **74**, 18-26 (2019). <https://doi.org/10.1016/j.ijdevneu.2019.02.005>

142 Amanzadeh Jajin, E., Esmaeili, A., Rahgozar, S. & Noorbakhshnia, M. Quercetin-conjugated superparamagnetic iron oxide nanoparticles protect AlCl3-induced neurotoxicity in a rat model of Alzheimer’s disease via antioxidant genes, APP gene, and miRNA-101. *Frontiers in Neuroscience* **14**, 598617 (2021).

143 Zhou, X., Li, G., Yang, B. & Wu, J. Quercetin Enhances Inhibitory Synaptic Inputs and Reduces Excitatory Synaptic Inputs to OFF- and ON-Type Retinal Ganglion Cells in a Chronic Glaucoma Rat Model. *Front Neurosci* **13**, 672 (2019). <https://doi.org/10.3389/fnins.2019.00672>

144 Liu, Y. *et al.* Quercetin-modified gold-palladium nanoparticles as a potential autophagy inducer for the treatment of Alzheimer's disease. *J Colloid Interface Sci* **552**, 388-400 (2019). <https://doi.org/10.1016/j.jcis.2019.05.066>

145 Guo, B., Zhang, Y., Hui, Q., Wang, H. & Tao, K. Naringin suppresses the metabolism of A375 cells by inhibiting the phosphorylation of c-Src. *Tumour Biol* **37**, 3841-3850 (2016). <https://doi.org/10.1007/s13277-015-4235-z>

146 Aslan, E., Guler, C. & Adem, S. In vitro effects of some flavonoids and phenolic acids on human pyruvate kinase isoenzyme M2. *J Enzyme Inhib Med Chem* **31**, 314-317 (2016). <https://doi.org/10.3109/14756366.2015.1022173>

147 Zhou, T., Liu, L., Wang, Q. & Gao, Y. Naringenin alleviates cognition deficits in high-fat diet-fed SAMP8 mice. *J Food Biochem* **44**, e13375 (2020). <https://doi.org/10.1111/jfbc.13375>

148 Qiu, Q. *et al.* Naringin Protects against Tau Hyperphosphorylation in Aβ25–35‐Injured PC12 Cells through Modulation of ER, PI3K/AKT, and GSK‐3β Signaling Pathways. *Behavioural Neurology* **2023**, 1857330 (2023).

149 Varshney, V. & Garabadu, D. Naringin Exhibits Mas Receptor-Mediated Neuroprotection Against Amyloid Beta-Induced Cognitive Deficits and Mitochondrial Toxicity in Rat Brain. *Neurotox Res* **39**, 1023-1043 (2021). <https://doi.org/10.1007/s12640-021-00336-y>

150 Zhu, Y. *et al.* Naringenin ameliorates amyloid-beta pathology and neuroinflammation in Alzheimer's disease. *Commun Biol* **7**, 912 (2024). <https://doi.org/10.1038/s42003-024-06615-6>

151 Salehpour, M., Ashabi, G., Kashef, M., Marashi, E. S. & Ghasemi, T. Aerobic Training with Naringin Supplementation Improved Spatial Cognition via H(2)S Signaling Pathway in Alzheimer's Disease Model Rats. *Exp Aging Res* **49**, 407-420 (2023). <https://doi.org/10.1080/0361073X.2022.2101303>

152 Kusi, M., Becer, E., Vatansever, H. S. & Yucecan, S. Neuroprotective Effects of Hesperidin and Naringin in SK-N-AS Cell as an In Vitro Model for Alzheimer's Disease. *J Am Nutr Assoc* **42**, 418-426 (2023). <https://doi.org/10.1080/07315724.2022.2062488>

153 Dashputre, N. L. *et al.* Potential therapeutic effects of naringin loaded PLGA nanoparticles for the management of Alzheimer's disease: In vitro, ex vivo and in vivo investigation. *Heliyon* **9**, e19374 (2023). <https://doi.org/10.1016/j.heliyon.2023.e19374>

154 Hassan, H. M. *et al.* Neuroprotective effect of naringin against cerebellar changes in Alzheimer’s disease through modulation of autophagy, oxidative stress and tau expression: An experimental study. *Frontiers in Neuroanatomy* **16**, 1012422 (2022).

155 Choi, G. Y. *et al.* Naringin enhances long-term potentiation and recovers learning and memory deficits of amyloid-beta induced Alzheimer's disease-like behavioral rat model. *Neurotoxicology* **95**, 35-45 (2023). <https://doi.org/10.1016/j.neuro.2022.12.007>

156 Meng, X. *et al.* Naringin ameliorates memory deficits and exerts neuroprotective effects in a mouse model of Alzheimer's disease by regulating multiple metabolic pathways. *Mol Med Rep* **23** (2021). <https://doi.org/10.3892/mmr.2021.11971>

157 Yan, S. H. *et al.* Chemoproteomics reveals berberine directly binds to PKM2 to inhibit the progression of colorectal cancer. *iScience* **25**, 104773 (2022). <https://doi.org/10.1016/j.isci.2022.104773>

158 Sun, Z. *et al.* Berberine Targets PKM2 to Activate the t-PA-Induced Fibrinolytic System and Improves Thrombosis. *Pharmaceuticals (Basel)* **17** (2024). <https://doi.org/10.3390/ph17091219>

159 Li, Z., Li, H., Lu, Y., Yang, P. & Li, Z. Berberine inhibited the proliferation of cancer cells by suppressing the activity of tumor pyruvate kinase M2. *Natural Product Communications* **12**, 1934578X1701200909 (2017).

160 Chen, L. & Mao, Y. Berberine inhibits the glycolysis and proliferation of hepatocellular carcinoma cells by down-regulating HIF-1α. *Pakistan Journal of Pharmaceutical Sciences* **37** (2024).

161 Du, Y. *et al.* Berberine Attenuates Cell Motility via Inhibiting Inflammation-Mediated Lysyl Hydroxylase-2 and Glycolysis. *Frontiers in Pharmacology* **13**, 856777 (2022).

162 Durairajan, S. S. *et al.* Berberine ameliorates beta-amyloid pathology, gliosis, and cognitive impairment in an Alzheimer's disease transgenic mouse model. *Neurobiol Aging* **33**, 2903-2919 (2012). <https://doi.org/10.1016/j.neurobiolaging.2012.02.016>

163 Huang, M. *et al.* Berberine improves cognitive impairment by promoting autophagic clearance and inhibiting production of beta-amyloid in APP/tau/PS1 mouse model of Alzheimer's disease. *Exp Gerontol* **91**, 25-33 (2017). <https://doi.org/10.1016/j.exger.2017.02.004>

164 Liang, Y. *et al.* Berberine Improves Behavioral and Cognitive Deficits in a Mouse Model of Alzheimer's Disease via Regulation of beta-Amyloid Production and Endoplasmic Reticulum Stress. *ACS Chem Neurosci* **12**, 1894-1904 (2021). <https://doi.org/10.1021/acschemneuro.0c00808>

165 Chen, Y. *et al.* Corrigendum to "Berberine mitigates cognitive decline in an Alzheimer's Disease Mouse Model by targeting both tau hyperphosphorylation and autophagic clearance" [Biomed. Pharmacother. 121 (2020) 109670]. *Biomed Pharmacother* **178**, 117250 (2024). <https://doi.org/10.1016/j.biopha.2024.117250>

166 Lin, L. *et al.* Synergic effects of berberine and curcumin on improving cognitive function in an Alzheimer’s disease mouse model. *Neurochemical research* **45**, 1130-1141 (2020).

167 Ye, C. *et al.* Berberine Improves Cognitive Impairment by Simultaneously Impacting Cerebral Blood Flow and beta-Amyloid Accumulation in an APP/tau/PS1 Mouse Model of Alzheimer's Disease. *Cells* **10** (2021). <https://doi.org/10.3390/cells10051161>

168 Chen, M., Li, L., Liu, C. & Song, L. Berberine attenuates Abeta-induced neuronal damage through regulating miR-188/NOS1 in Alzheimer's disease. *Mol Cell Biochem* **474**, 285-294 (2020). <https://doi.org/10.1007/s11010-020-03852-1>

169 Xuan, W. T. *et al.* Berberine ameliorates rats model of combined Alzheimer's disease and type 2 diabetes mellitus via the suppression of endoplasmic reticulum stress. *3 Biotech* **10**, 359 (2020). <https://doi.org/10.1007/s13205-020-02354-7>

170 Jia, L. *et al.* Berberine suppresses amyloid-beta-induced inflammatory response in microglia by inhibiting nuclear factor-kappaB and mitogen-activated protein kinase signalling pathways. *J Pharm Pharmacol* **64**, 1510-1521 (2012). <https://doi.org/10.1111/j.2042-7158.2012.01529.x>

171 Zhang, H. *et al.* Berberine modulates amyloid-beta peptide generation by activating AMP-activated protein kinase. *Neuropharmacology* **125**, 408-417 (2017). <https://doi.org/10.1016/j.neuropharm.2017.08.013>

172 Cai, Z., Wang, C., He, W. & Chen, Y. Berberine Alleviates Amyloid-Beta Pathology in the Brain of APP/PS1 Transgenic Mice via Inhibiting beta/gamma-Secretases Activity and Enhancing alpha-Secretases. *Curr Alzheimer Res* **15**, 1045-1052 (2018). <https://doi.org/10.2174/1567205015666180702105740>

173 He, W., Wang, C., Chen, Y., He, Y. & Cai, Z. Berberine attenuates cognitive impairment and ameliorates tau hyperphosphorylation by limiting the self-perpetuating pathogenic cycle between NF-kappaB signaling, oxidative stress and neuroinflammation. *Pharmacol Rep* **69**, 1341-1348 (2017). <https://doi.org/10.1016/j.pharep.2017.06.006>

174 Wu, Y. *et al.* Berberine reduces Aβ42 deposition and tau hyperphosphorylation via ameliorating endoplasmic reticulum stress. *Frontiers in Pharmacology* **12**, 640758 (2021).

175 Li, S. *et al.* Genistein suppresses aerobic glycolysis and induces hepatocellular carcinoma cell death. *British journal of cancer* **117**, 1518-1528 (2017).

176 Gao, H. *et al.* Corrigendum to "Genistein attenuates memory impairment in Alzheimer's disease via ERS-mediated apoptotic pathway in vivo and in vitro" [The Journal of Nutritional Biochemistry Volume 109 (2022) 109118]. *J Nutr Biochem* **131**, 109710 (2024). <https://doi.org/10.1016/j.jnutbio.2024.109710>

177 Petry, F. D. S. *et al.* Genistein protects against amyloid-beta-induced toxicity in SH-SY5Y cells by regulation of Akt and Tau phosphorylation. *Phytother Res* **34**, 796-807 (2020). <https://doi.org/10.1002/ptr.6560>

178 Park, Y. J., Ko, J. W., Jeon, S. & Kwon, Y. H. Protective Effect of Genistein against Neuronal Degeneration in ApoE(-/-) Mice Fed a High-Fat Diet. *Nutrients* **8** (2016). <https://doi.org/10.3390/nu8110692>

179 Petry, F. D. S. *et al.* Genistein attenuates amyloid-beta-induced cognitive impairment in rats by modulation of hippocampal synaptotoxicity and hyperphosphorylation of Tau. *J Nutr Biochem* **87**, 108525 (2021). <https://doi.org/10.1016/j.jnutbio.2020.108525>

180 Bagheri, M., Roghani, M., Joghataei, M. T. & Mohseni, S. Genistein inhibits aggregation of exogenous amyloid-beta(1)(-)(4)(0) and alleviates astrogliosis in the hippocampus of rats. *Brain Res* **1429**, 145-154 (2012). <https://doi.org/10.1016/j.brainres.2011.10.020>

181 Ye, S. *et al.* Genistein protects hippocampal neurons against injury by regulating calcium/calmodulin dependent protein kinase IV protein levels in Alzheimer's disease model rats. *Neural Regen Res* **12**, 1479-1484 (2017). <https://doi.org/10.4103/1673-5374.215260>

182 Wang, Y. *et al.* Genistein suppresses the mitochondrial apoptotic pathway in hippocampal neurons in rats with Alzheimer's disease. *Neural Regen Res* **11**, 1153-1158 (2016). <https://doi.org/10.4103/1673-5374.187056>

183 Vina, J. *et al.* Genistein effect on cognition in prodromal Alzheimer's disease patients. The GENIAL clinical trial. *Alzheimers Res Ther* **14**, 164 (2022). <https://doi.org/10.1186/s13195-022-01097-2>

184 Valles, S. L. *et al.* Estradiol or genistein prevent Alzheimer's disease-associated inflammation correlating with an increase PPARγ expression in cultured astrocytes. *Brain research* **1312**, 138-144 (2010).

185 Pierzynowska, K. *et al.* Autophagy-dependent mechanism of genistein-mediated elimination of behavioral and biochemical defects in the rat model of sporadic Alzheimer's disease. *Neuropharmacology* **148**, 332-346 (2019). <https://doi.org/10.1016/j.neuropharm.2019.01.030>

186 Wu, Q. *et al.* Caffeic acid phenethyl ester inhibits MDA-MB-231 cell proliferation in inflammatory microenvironment by suppressing glycolysis and lipid metabolism. *Biomed Pharmacother* **168**, 115766 (2023). <https://doi.org/10.1016/j.biopha.2023.115766>

187 Zhang, Y. *et al.* Caffeic acid reduces A53T alpha-synuclein by activating JNK/Bcl-2-mediated autophagy in vitro and improves behaviour and protects dopaminergic neurons in a mouse model of Parkinson's disease. *Pharmacol Res* **150**, 104538 (2019). <https://doi.org/10.1016/j.phrs.2019.104538>

188 Sun, R. *et al.* Caffeic acid protects against atherosclerotic lesions and cognitive decline in ApoE(-/-) mice. *J Pharmacol Sci* **151**, 110-118 (2023). <https://doi.org/10.1016/j.jphs.2022.12.006>

189 Andrade, S., Loureiro, J. A. & Pereira, M. C. Caffeic acid for the prevention and treatment of Alzheimer's disease: The effect of lipid membranes on the inhibition of aggregation and disruption of Abeta fibrils. *Int J Biol Macromol* **190**, 853-861 (2021). <https://doi.org/10.1016/j.ijbiomac.2021.08.198>

190 Chang, W. *et al.* Protective Effect of Caffeic Acid against Alzheimer's Disease Pathogenesis via Modulating Cerebral Insulin Signaling, beta-Amyloid Accumulation, and Synaptic Plasticity in Hyperinsulinemic Rats. *J Agric Food Chem* **67**, 7684-7693 (2019). <https://doi.org/10.1021/acs.jafc.9b02078>

191 Ding, Z. *et al.* Emodin ameliorates antioxidant capacity and exerts neuroprotective effect via PKM2-mediated Nrf2 transactivation. *Food and Chemical Toxicology* **160**, 112790 (2022).

192 Xing, Y.-X. *et al.* Anti-cancer effects of emodin on HepG2 cells as revealed by 1H NMR based metabolic profiling. *Journal of proteome research* **17**, 1943-1952 (2018).

193 Zhang, F.-Y. *et al.* Emodin induces apoptosis and suppresses non-small-cell lung cancer growth via downregulation of sPLA2-IIa. *Phytomedicine* **95**, 153786 (2022).

194 Du, C. *et al.* Emodin attenuates Alzheimer's disease by activating the protein kinase C signaling pathway. *Cell Mol Biol (Noisy-le-grand)* **65**, 32-37 (2019).

195 Zeng, P. *et al.* Emodin Rescued Hyperhomocysteinemia-Induced Dementia and Alzheimer's Disease-Like Features in Rats. *Int J Neuropsychopharmacol* **22**, 57-70 (2019). <https://doi.org/10.1093/ijnp/pyy090>

196 Li, Z. *et al.* [Corrigendum] Neuroprotective effect of emodin against Alzheimer's disease via Nrf2 signaling in U251 cells and APP/PS1 mice. *Mol Med Rep* **27** (2023). <https://doi.org/10.3892/mmr.2023.12994>

197 Wang, L. *et al.* Emodin inhibits aggregation of amyloid-beta peptide 1-42 and improves cognitive deficits in Alzheimer's disease transgenic mice. *J Neurochem* **157**, 1992-2007 (2021). <https://doi.org/10.1111/jnc.15156>

198 Zhao, X. *et al.* Ameliorating Effects of Aloe Emodin in an Aluminum‐Induced Alzheimer’s Disease Rat Model. *Journal of Food Biochemistry* **2024**, 7306081 (2024).

199 Xia, N. *et al.* Neuroprotective effect of emodin on Aβ25-35-induced cytotoxicity in PC12 cells involves Nrf2/GPX4 and TLR4/p-NF-κB/NLRP3 pathways. *Brain Research*, 149019 (2024).

200 Ding, Z. *et al.* Emodin ameliorates antioxidant capacity and exerts neuroprotective effect via PKM2-mediated Nrf2 transactivation. *Food Chem Toxicol* **160**, 112790 (2022). <https://doi.org/10.1016/j.fct.2021.112790>

201 Jiang, W. *et al.* Neuroprotection of Emodin by Inhibition of Microglial NLRP3 Inflammasome-Mediated Pyroptosis. *J Integr Neurosci* **22**, 48 (2023). <https://doi.org/10.31083/j.jin2202048>

202 Luo, P. *et al.* Celastrol mitigates inflammation in sepsis by inhibiting the PKM2-dependent Warburg effect. *Military Medical Research* **9**, 22 (2022).

203 Fan, N. *et al.* Covalent inhibition of pyruvate kinase M2 reprograms metabolic and inflammatory pathways in hepatic macrophages against non-alcoholic fatty liver disease. *International journal of biological sciences* **18**, 5260 (2022).

204 Liang, L.-J. *et al.* CIP2A induces PKM2 tetramer formation and oxidative phosphorylation in non-small cell lung cancer. *Cell Discovery* **10**, 13 (2024).

205 Yang, C. *et al.* Celastrol enhances transcription factor EB (TFEB)-mediated autophagy and mitigates Tau pathology: Implications for Alzheimer's disease therapy. *Acta Pharm Sin B* **12**, 1707-1722 (2022). <https://doi.org/10.1016/j.apsb.2022.01.017>

206 Zhang, C. *et al.* The Nrf2-NLRP3-caspase-1 axis mediates the neuroprotective effects of Celastrol in Parkinson's disease. *Redox Biol* **47**, 102134 (2021). <https://doi.org/10.1016/j.redox.2021.102134>

207 Anastasiou, D. *et al.* Pyruvate kinase M2 activators promote tetramer formation and suppress tumorigenesis. *Nat Chem Biol* **8**, 839-847 (2012). <https://doi.org/10.1038/nchembio.1060>

208 Yi, Z. *et al.* Activator-Mediated Pyruvate Kinase M2 Activation Contributes to Endotoxin Tolerance by Promoting Mitochondrial Biogenesis. *Front Immunol* **11**, 595316 (2020). <https://doi.org/10.3389/fimmu.2020.595316>

209 Yang, L. *et al.* Upregulated expression of ubiquitin ligase TRIM21 promotes PKM2 nuclear translocation and astrocyte activation in experimental autoimmune encephalomyelitis. *Elife* **13** (2024). <https://doi.org/10.7554/eLife.98181>

210 Han, J. *et al.* Aberrant role of pyruvate kinase M2 in the regulation of gamma-secretase and memory deficits in Alzheimer's disease. *Cell Rep* **37**, 110102 (2021). <https://doi.org/10.1016/j.celrep.2021.110102>

211 Li, M. *et al.* Pyruvate kinase M2 (PKM2) interacts with activating transcription factor 2 (ATF2) to bridge glycolysis and pyroptosis in microglia. *Mol Immunol* **140**, 250-266 (2021). <https://doi.org/10.1016/j.molimm.2021.10.017>

212 Gao, S. *et al.* PKM2 promotes pulmonary fibrosis by stabilizing TGF-beta1 receptor I and enhancing TGF-beta1 signaling. *Sci Adv* **8**, eabo0987 (2022). <https://doi.org/10.1126/sciadv.abo0987>

213 Park, J. H. *et al.* Specific Pyruvate Kinase M2 Inhibitor, Compound 3K, Induces Autophagic Cell Death through Disruption of the Glycolysis Pathway in Ovarian Cancer Cells. *Int J Biol Sci* **17**, 1895-1908 (2021). <https://doi.org/10.7150/ijbs.59855>

214 Yang, Y. *et al.* Lapachol treats non-alcoholic fatty liver disease by modulating the M1 polarization of Kupffer cells via PKM2. *Int Immunopharmacol* **120**, 110380 (2023). <https://doi.org/10.1016/j.intimp.2023.110380>

215 Pan, R. Y. *et al.* Positive feedback regulation of microglial glucose metabolism by histone H4 lysine 12 lactylation in Alzheimer's disease. *Cell Metab* **34**, 634-648 e636 (2022). <https://doi.org/10.1016/j.cmet.2022.02.013>

216 Wei, X. *et al.* Platelet-rich plasma improves chronic inflammatory pain by inhibiting PKM2-mediated aerobic glycolysis in astrocytes. *Ann Transl Med* **8**, 1456 (2020). <https://doi.org/10.21037/atm-20-6502>

217 Zhu, H. *et al.* Tetramerization of PKM2 Alleviates Traumatic Brain Injury by Ameliorating Mitochondrial Damage in Microglia. *J Neuroimmune Pharmacol* **19**, 48 (2024). <https://doi.org/10.1007/s11481-024-10138-6>
